# Molecular Dynamics Reveals Unique Distal Histidine Positions and Interfacial Transitions of Fish and Mammalian Deoxyhemoglobin at Post-mortem pH

**DOI:** 10.1021/acs.jafc.5c05805

**Published:** 2025-08-08

**Authors:** Sean Baker, Mark P. Richards

**Affiliations:** † Department of Food Science, 5228 University of Wisconsin−Madison, Madison, Wisconsin 53706, United States; ‡ Department of Animal and Dairy Sciences, University of Wisconsin–Madison, Madison, Wisconsin 53706, United States

**Keywords:** root effect, lipid oxidation, molecular dynamics, hemoglobin

## Abstract

Molecular dynamics simulations were utilized to evaluate the mechanisms that cause trout IV Hb to have enhanced oxidative capacity compared to bovine Hb. The focal points of analysis were amino acid differences in the heme pocket and at the α_1_–β_2_ interface and the effect of varying distal histidine protonation state. Unprotonated HisE7 that swung up came back down only in bovine Hb, attributable to weaker π–π stacking interactions for PheCE4 (bovine) compared to TrpCE4 (trout IV). Protonation of HisE7 causes this residue to stay pointed down toward the heme only in bovine DHb. This finding was complimented by experimental findings indicating elevated nitrite reductase activity of bovine DHb at low pH, highlighting the importance of the HisE7 orientation for catalysis. Computational analysis indicated key interfacial differences that may promote tetramer disassembly of fish DHb at a low pH. Size exclusion chromatography data corroborated this finding, showing a differential size distribution between fish and mammalian DHb at pH 5.7. Overall, these studies suggest that the elevated pro-oxidative capacity of trout IV Hb is related to an increased tendency for subunit formation and a greater propensity for HisE7 to be in the “up” position providing a channel for oxidants to enter the heme pocket.

## Graphical abstract

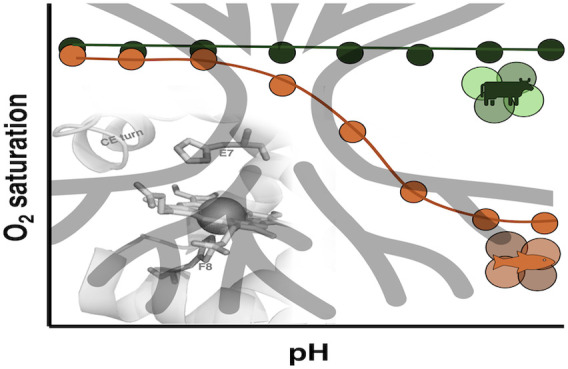

## Introduction

Hemoglobin (Hb)-mediated lipid peroxidation is a major contributor to color deterioration^1^ and off-odor formation,^2^ thus leading to decreased consumer acceptability of muscle foods. In addition to quality defects, research has shown that potentially cytotoxic (4-hydroxy-trans-2-nonenal) and mutagenic (malondialdehyde) secondary lipid peroxidation products have been detected in muscle foods,^3,4^ compounding on the observed quality defects. A major contributor to organoleptic defects of muscle foods is the release of pro-oxidant hemin from the globin of Hb, which then partitions into lipid membranes^5^ to facilitate peroxidation. The events preceding and the hemin release event itself are complicated and impacted by the Hb amino acid sequence,^6^ system pH,^7^ and protein–protein interactions,^8^ among other factors. Aranda and others^6^ did groundbreaking work to solve the crystal structures of fish (i.e., trout IV and perch) and mammalian (i.e., bovine) Hb at various pH values allowing future computational research on how amino acid sequence and pH may impact the global/local structure of Hb from different species. Herein, we will focus on the comparison between bovine and trout IV Hb, which share 50% and 48% α-chain and β-chain sequence homology, respectively. The interest in these two species lies in their value as agricultural commodities^9,10^ and the stark contrast in their functional characteristics, particularly at post-mortem pH. Trout IV displays rapid auto-oxidation and a very strong Root effect (Hb deoxygenation at low pH), which contrasts strongly with bovine Hb.^6^ Previous research has performed molecular dynamics (MD) simulations focused on the heme environment of human hemoglobin in its deoxy (Fe^2+^)^11^ and met (Fe^3+^) forms.^12^ The former studied the binding of additional heme by DHb to model in vivo hemolytic conditions, while the latter probed hemin dissociation by manually breaking the proximal histidine–iron bond. To date, no MD simulations have been performed to probe the heme pocket and global structural transitions from the Hb of valuable agricultural commodities. Further, it has been proposed that distal histidine protonation is a critical step in the hemoglobin-mediated lipid oxidation pathway.^7^ Our investigation aims to assess how the heme pocket and global structure differ between bovine and trout IV deoxyhemoglobin (DHb) when the α-chain distal histidine is in its neutral and doubly protonated state. This work employs MD calculations combined with biochemical assays (DHb pseudoenzymatic activity and size exclusion chromatography (SEC)) to compliment MD findings.

## Methods

### Model Preparation and Simulation

Using CHARMM-GUI trout IV (PDB: 3BOM) and bovine (PDB: 2QSP) Hb were prepared for multicomponent assembly using PDB Reader & Manipulator,^13–15^ in which the pH was set to 5.7, with crystal waters included and crystallization aids removed (i.e., ethanediol). Native bovine and trout IV Hb contained 10 and 6 protonated sites, respectively, at pH 5.7 (Tables S1 and S2). Protonation states were determined at pH 5.7 using PROPKA3.^16^ On the α-chains, protonation was performed on the distal histidine (HisE7) resulting in a proton on N_δ_ and N_ε_ (HSP) (α-prot). In contrast, the β-chain E7 residues were kept in the deprotonated state, with only the N_δ_ bearing a proton (HSD). We focused on the α-chain protonation due to experimental evidence suggesting profound increases in auto-oxidation when Ile is found at the E11 position, which is the case for trout IV Hb.^17^ This suggests a more pro-oxidative character of the α-chain. An unmodified PDB was used as the control, where the neutral HisE7 had a proton on N_δ_ (π-tautomer, HSD) for the α and β chains (α-native) (Figure S1A). The HSD tautomer was selected based on previous research indicating a nearly 1:1 ratio of HisE7 tautomer’s in horse myoglobin, as measured by covalent labeling.^18^ For all chains, heme coordination occurred with the proximal histidine residue (F8). The .psf and .crd files from the PDB reader were input into the CHARM-GUI Multicomponent Assembler.^13,19^ One hemoglobin molecule was solvated in a box with dimensions of 10 × 10 × 10 nm. The solvent was composed of water (TIP3P water model, *n* = 28,757–28,803) and Na^+^ (*n* = 15–17) and PO_4_^3–^ (*n* = 5) ions (placing method: distance). A CHARMM36m^20–22^ force field with hydrogen mass partitioning^23^ was employed. This force field assigned charges of 0.24 for Fe and −0.18 for the 4 coordinating porphyrin N atoms. The default CHARMM36m Fe parameters were used (*toppar_all36_prot_heme.str*), leading to a 5-bond (4 pyrrole N, 1 proximal histidine N_ε_) Fe coordination scheme. These assigned charges align with a ferrous heme model with a water molecule as the sixth ligand;^24^ thus, it is said that our starting models are deoxy, ferrous Hb with a water in the distal cavity. As far as defining the global structure, our model follows the early stage (first ∼150 ns) of an R-like → T transition.^25,26^ [Fn fn1] Energy minimization was performed using the steepest descent algorithm for 5000 steps, followed by equilibration for 125 ps using an *NVT* ensemble at 298 K equipped with a Nose–Hoover thermocouple (coupling constant of 1 ps) and an integrator. Production simulation was then performed with an *NPT* ensemble at 298 K using a Nose–Hoover thermocouple and a Parrinello–Rahman pressure couple (coupling constant of 5 ps). Simulations were repeated multiple times (*n* = 5), with random velocity generation. The constraint algorithm for all steps was LINCS with constraints set to H-bonds. The minimization and equilibration code was executed in the jupyter notebook using GROMACS v2024.2.^27^

In silico mutations were performed in PyMol, and then, the model was subject to identical model preparation as described above. However, the production simulation was performed using the V-rescale and C-rescale thermocouple and pressure couple, respectively, among other minor modifications (see the Supporting Information for parameters).

Production simulations were performed using the high-performance computing resources at the Center for High Throughput Computing (CHTC UW Madison). Simulations were allowed to run for 24 h using 4 nodes with 64 cores/node. Due to computing resource efficiency discrepancies, the simulation time (ns) was not identical for all models (Table S3). Simulation visualization was performed in PyMol v2.5.2. Complete minimization, equilibration, and production inputs can be viewed in the Supporting Information. All models and trajectory files for all simulation replicates can be downloaded from the ScienceDB repository (https://www.scidb.cn/en/s/mAbmUv).

### Simulation Postprocessing

Upon completion of the MD simulation, all data past 1 ns was analyzed using GROMACS post-processing packages. Because no *NPT* equilibration was performed, it was verified that the pressure and volume for all constructs converged within 1 ns (Figure S1B–E). The energy (*gmx energy*) driven by the additive potential energy function (eqs S1–S3) and rms (*gmx rms*) functions were used to confirm simulation quality. Index files were made for the N_ε_ of His E7 and heme iron for all chains using the *make_ndx* function, and then distances (*gmx distance*) between the atoms were output as .xvg files using the distance function (Δ*t* = 0.2 ns). Indexing and distance calculations were repeated for the N_ε_ of His F8 distance to the heme iron. Regarding the heme iron, we also measured the displacement (*gmx distance*) of the heme iron from the heme plane of the α-chains. This was performed by indexing the four pyrrole nitrogen’s, then measuring the distance between the iron atom and the center of mass of the pyrrole nitrogen’s. Further, α_1_–β_2_ interfacial interactions were analyzed using the index and distance functions, as described above. Interfacial residues were identified based on the previous literature, outlining residue-specific interactions of hemoglobin α_1_ and β_2_ chains.^12^ Interactions of interest were hydrogen bonding Asp­(α_1_G1) CG → Asn­(β_2_G4) ND2 (bovine and trout), stacking Tyr­(α_1_C7) CZ → Arg­(β_2_C6) CZ (bovine and trout), stacking Arg­(α_1_FG4) CD → Arg­(β_2_C6) CB (bovine and trout), and hydrogen bonding Thr­(α_1_C6) OG → Arg­(β_2_C6) CZ (bovine only). In addition to global backbone RMSD (*gmx rms*), local RMSD (*gmx rms*) of the α_1_β_2_ interface was also performed. The backbone atoms (N, CA, and O, 48 atoms/group) of α_1_C6, α_1_C7, α_1_FG4, α_1_G1, β_2_C6, and β_2_G4 were indexed for bovine and trout IV DHb in their native and α-prot states. This index was then used to calculate the backbone RMSD of the interface. Heme pocket solvent access was assessed by calculating the solvent-accessible surface area (SASA, *gmx sasa*)^28,29^ of residue E11 and the heme moiety in the α-chains. This was performed by indexing the heme atoms with the protein atoms, followed by SASA analysis. The average SASA for residue E11 was calculated for all simulations from 1 to 133 ns. A complete list of selected atoms for post-processing analysis can be seen in Tables S4 and S5.

### p*K*_a_ Calculations

p*K*_a_ calculations were performed using a Poisson–Boltzmann-based module, PypKa.^30^ PDB codes were used to input structures, and then, the p*K*_a_ of αHisE7 residues was computed using default parameters: pH range = 0–12, pH step = 0.25, protein dielectric = 15, solvent dielectric = 80, and ionic strength = 0.1. PypKa utilizes PDB 2PQR^31^ for determining titration states which does not recognize heme moieties or heme iron, so the p*K*_a_ values we have reported are calculated only considering globin amino acids.

### Hemoglobin Nitrite Reductase Activity

The nitrite reductase activity of trout IV and bovine DHb was performed following previously published procedures.^32–35^ Trout IV and bovine Hb were isolated from fresh blood according to previously published methods.^6^ 2 μL of enzyme solution (20 mg glucose oxidase/mL and 2 mg catalase/mL) was added to 1 mL of the reaction buffer (0.3% d-glucose in 10 mM MES pH 5.7 or 10 mM sodium phosphate pH 7.4) and allowed to incubate for 3 min, before the stepwise addition of (1) a few crystals of sodium dithionite,[Fn fn2] (2) 5 μL of 270 mM NaNO_2_ in Milli-Q water, and (3) 10 μL of 1 mM oxyHb stock solution. The solution was mixed rapidly and held at room temperature (22 °C) for the duration of the reaction. Electronic absorption spectra (2 nm step) were collected every 30 s for 180 s using a Shimadzu UV-2600 dual beam spectrophotometer. Ahead of the reaction, all buffer and stock solutions were allowed to equilibrate to room temperature, followed by deoxygenation using a constant stream of argon for 40 min. Experiments were replicated 3–4 times.

### SEC of DHb

SEC was performed as outlined previously.^36^ Columns were self-packed with 15 mL of Sepharose CL-6B into a 20 mL chromatography column (Econo-Pac Chrom. Column, Bio-Rad, Hercules, CA). The resin was allowed to settle for 2 h, followed by the insertion of a frit at the top of the resin bed. The column was drained of the storage solution, followed by a rinse with 15 mL of Milli-Q water and then a rinse with 15 mL of 10 mM MES pH 5.7. 500 μL of DHb sample was then loaded onto the column and allowed to pass through the frit before the addition of 12 mL of elution buffer (10 mM MES pH 5.7). The eluent was collected in 500 μL fractions, which were then measured for their 280 nm absorbance (UV-2600, Shimadzu, Kyoto, Japan) in matched black cuvettes. The DHb that was loaded onto the column was 40 μM Hb in 10 mM MES pH 5.7 with 5 mg of sodium dithionite and was used immediately. All buffers were extensively flushed with argon prior to use, and there was a continuous flow of argon down the SEC column during column preparation and sample separation. All separations were performed in triplicate on unique columns. The final data was baseline-corrected, retention time aligned,[Fn fn3] and then fit to a Gaussian distribution.

### Statistical Analysis

All data analysis was performed after 1 ns to allow for system equilibration (Figure S1B–E). Swing out events were defined as when the HisE7 N_ε_–Fe distance was greater than 7 Å. Values for the α chains were combined between the data for α_1_ and α_2_ (*n* = 10). To account for differences in simulation time length, the number of swing out events was corrected for the total simulation time (sum of all replicates) for a given treatment. Swing out time (time until HisE7 N_ε_–Fe > 7 Å) was collected from the distance between N_ε_ and heme iron data. Normalized swing out time was reported only if an event occurred. Replicates where no transition occurred were not included in the normalized swing out time calculation. Values for the α chains were combined between the data for α_1_ and α_2_. Again, to account for differences in simulation length, the swing out time was normalized to the longest simulations time for differences in simulation length within the group and total simulation time between the group. HisF8 N_ε_ to heme iron average distance values for the α chains were combined for α_1_ and α_2_ (*n* = 10) and β_1_ and β_2_ (*n* = 10). Each data point was the average length from 1 to 133 ns. Statistics were performed using a 1-way ANOVA with multiple comparisons. Iron displacement from the α-chain heme plane analysis was performed averaging the displacement across all data points from 1 to 133 ns (6610 data points/treatment). Statistics were performed using a 1-way ANOVA with multiple comparisons. Interfacial analysis was performed by collecting the maximum distance between selected atoms at a given interface site throughout the duration of the simulation (*n* = 5). Statistics were performed using 1-way ANOVA with multiple comparisons. Interfacial RMSD data was plotted between 1 and 133 ns for all replicates and fit to a simple linear regression. SASA analysis of residue E11 was performed by taking the average SASA value of residue E11 from all α-chains throughout the simulation (1–133 ns). Significance was calculated using an unpaired *t*-test within species. Due to differences in E11 identity, direct comparisons between species were not performed. Heme SASA was analyzed by 1-way ANOVA with multiple comparisons. All data was analyzed using GraphPad Prism v 0.1.1.

## Results and Discussion

### Deprotonated HisE7 Swings Away from the Heme Iron in Bovine and Trout IV DHb

Previous research has shown that hemoglobin (Hb) HisE7 can swing out away from the heme iron independent of iron redox status and sixth ligand identity.^6,37,38^ Our results show 8 and 4 instances of HSD swing out events (Fe–N_ε_ distance >7 Å) in native[Fn fn4] bovine (Figures 1A and 2B) and trout IV DHb (Figures 1B and 2E), respectively, repositioning itself toward the CE turn (Figure 2A). Bovine and trout IV share PheCE1 and HisCE3; however, bovine possesses a PheCE4 compared to the trout IV TrpCE4. The aromatic residues present at CE4 participate in T-shaped/parallel displaced (Figures 2H and S6) π–π stacking interactions and steric restriction^39^ that aid in stabilizing HisE7 in the up or down position. Particularly in the up position, HSD can be further stabilized through an H-bond with the carbonyl backbone of site E3 (Figure 2H). This will depend on the orientation of HSD during the swing out event. Because our data shows that HSD will swing out in both bovine and trout IV DHb, the CE4 identity becomes important as different stacking pairs have different interaction energies. Liao and others^40^ reported an elevated interaction energy when the stacking pair was imidazole-Trp compared to imidazole-Phe using both B3LYP and CCSD QM methods. Interestingly, among all the simulations performed for native bovine and native trout IV DHb, HSD returned to the down position on only 4 occasions, all in bovine DHb, possibly lending to the weaker interaction energy of HSD-Phe π–π interactions when HSD is in the up position.

**Figure 1:**
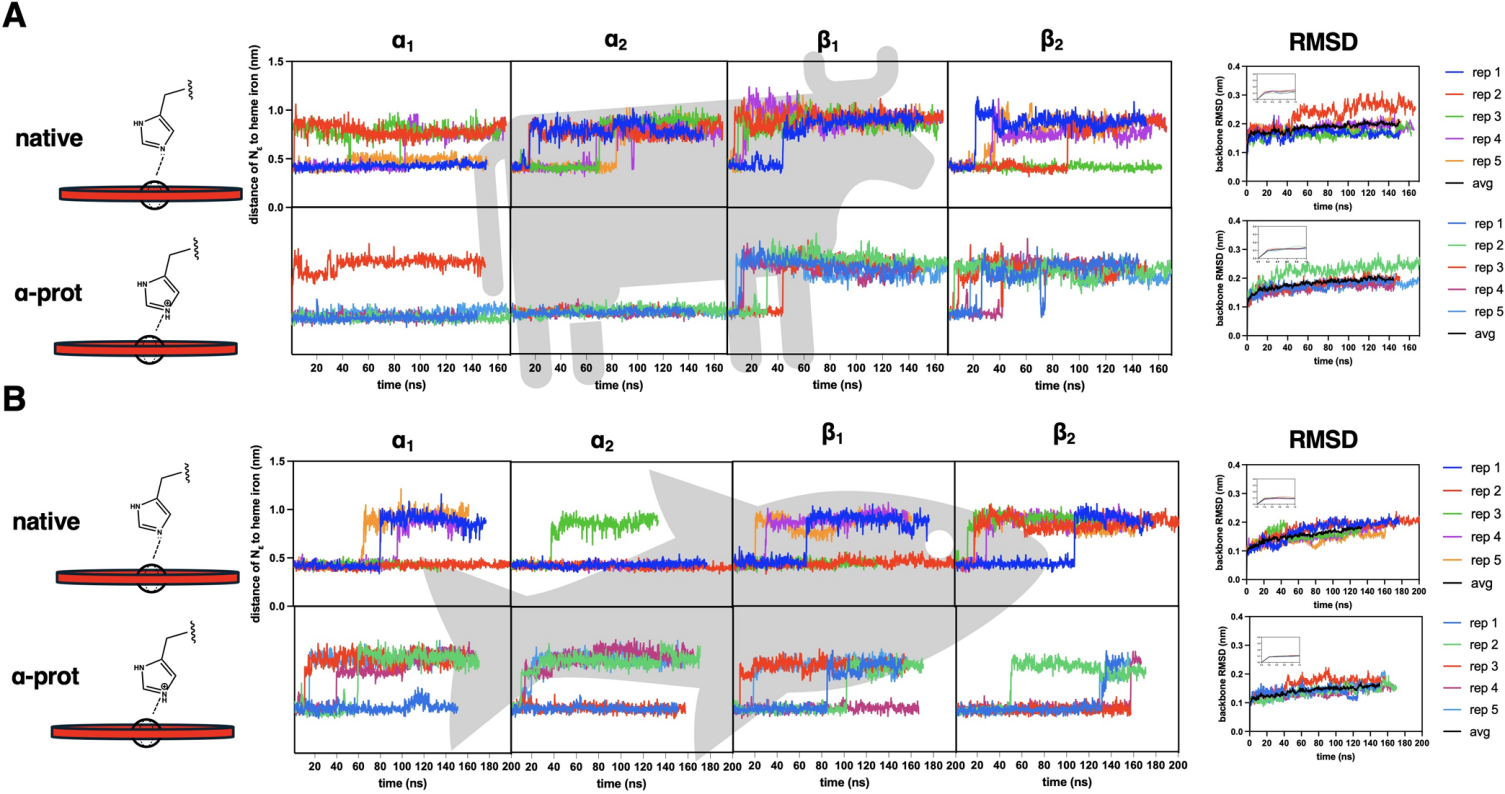
Distance between HisE7 N_ε_ and the heme iron and backbone RMSD. (A) Bovine deoxyhemoglobin (DHb), (top row) distance between HisE7 N_ε_ for all chains (bottom row) distance between HisE7 N_ε_ when the HisE7 of the α chains was in its protonated state (HSP), and the β HisE7 remained deprotonated. (B) Trout IV DHb, (top row) distance between HisE7 N_ε_ for all chains (bottom row) distance between HisE7 N_ε_ when the HisE7 of the α chains was in its protonated state, and the β HisE7 remained deprotonated.

**Figure 2:**
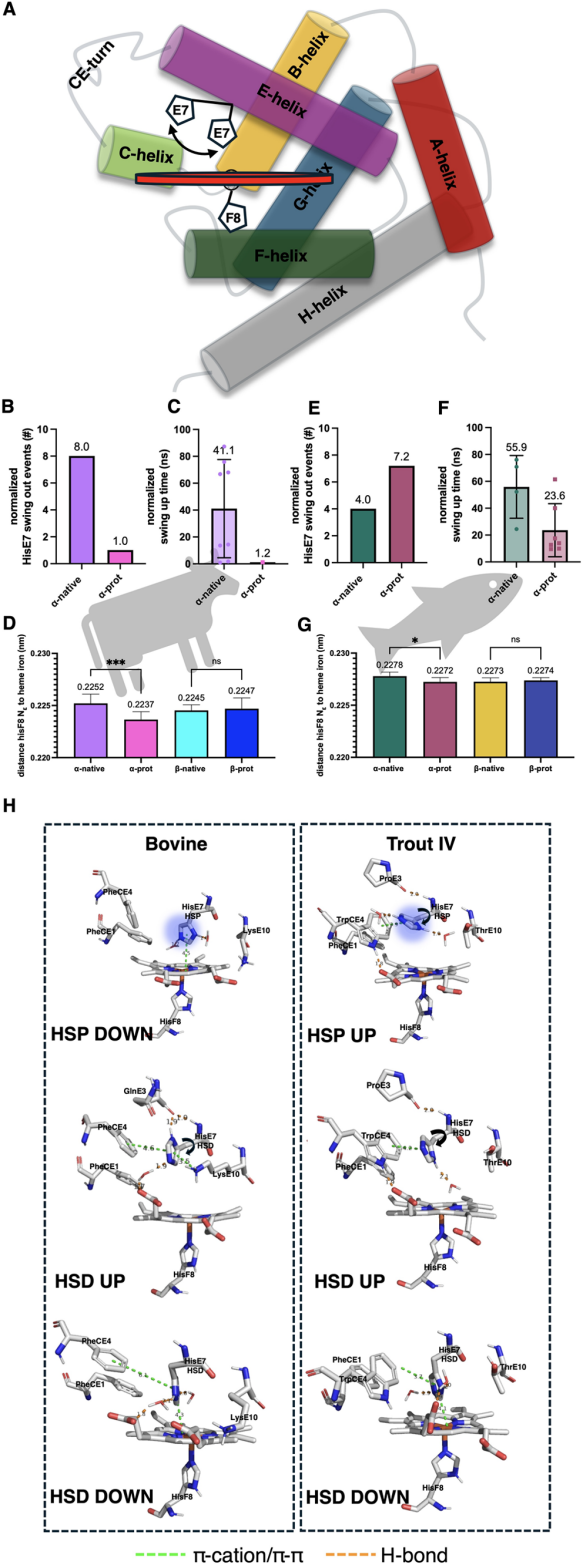
HisE7 position relative to the heme pocket of native α (α-native) and β (β-native) deoxyhemoglobin (DHb) and α chain with a protonated E7 histidine (α-prot). (A) Schematic of HisE7 swinging away from the heme pocket. (B) Normalized HisE7 swing out events of bovine DHb—the maximum number of events would be 10. (C) Normalized simulation time for HisE7 to swing out in bovine DHb α-native and α-prot. (D) Average distance between HisF8 N_ε_ and heme iron of bovine DHb. (E) Normalized HisE7 swing out events in trout IV DHb—the maximum number of events would be 10. (F) Simulation time for HisE7 to swing out in trout IV DHb α-native and α-prot. (G) Average distance between HisF8 N_ε_ and heme iron of trout IV DHb. (C/F) Normalized swing out time was collected from the distance between N_ε_ and heme iron data. Values for the α chains were combined between the data for α_1_ and α_2_ (*n* = 10). Normalization to the longest simulation was applied to account for differences in simulation time within treatment replicates and total simulation time between treatments. Reported data is the time it takes for the transition to occur. If no transition occurred, no data point was reported for that replicate. (D/G) Average distance values for the α chains were combined for α_1_ and α_2_ (*n* = 10) and β_1_ and β_2_ (*n* = 10). Each data point was the average length between 1 and 133 ns to ensure just comparisons. Statistics were performed using 1-way ANOVA with multiple comparisons. (H) Geometric analysis of distal HisE7 position for bovine DHb (left) and trout IV DHb (right). H-bond (orange) and π–π/π–cation/CH–π (green) interaction distances. HSP are displayed with the blue halo indicating positive charge. The π interactions involving the heme iron represent the distance between HisE7 and nearest cyclic portion of the heme. Nonpolar hydrogens have been removed for clarity.

### Protonated HisE7 Stays Down in Bovine But Not in Trout IV DHb

Upon protonation of HisE7 (HSP) in the α-chains of bovine DHb, this residue swung out on only 1 occasion, thus displaying far fewer swing out occurrences compared to the native bovine DHb where αHisE7 HSD swing out 8 times (Figure 2B). Further, the average normalized swing out time for HisE7 for α-natives was 41.1 ns (Figure 2C). In contrast, there was only one swing out transition occurring at 1.2 ns when αHisE7 was in the HSP (α-prot, Figure 2C). The trout IV diverged from the bovine in that there was an increase in swing out events (Figure 2E) and a decrease in the normalized swing out time following protonation, with α-native taking 55.9 ns and α-prot taking 23.6 ns on average (Figure 2F). The observed difference between trout and bovine may be attributed to the presence of TrpCE4 in trout IV DHb (PheCE4 in bovine). Previous research^40^ has shown that upon protonation of His, the π–π stacking interactions with Trp become more favorable by a factor of ∼12 and ∼2 when using B3LYP and CCSD QM methods, respectively. This is further corroborated by the findings of Calinsky and Levy,^41^ who show that the protonation of His creates a more favorable (−5.5 ± 1 kcal/mol) cation–π interaction with Trp, compared to a protonated His, cation–π interaction with Phe (−3.9 ± 0.6 kcal/mol).

Contrary to trout IV, the findings for bovine HSP seem anomalous considering the increased net charge on αHisE7 which is oriented toward the positively charged heme iron. The orientation of the HSP is in part stabilized by waters which fulfill the H-bonding potential of the HSP and a cation–π interaction where the HSP serves as the cation^41^ and the heme porphyrin ring as the π system^42^ (HSP C_ε_ is ∼4 Å from porphyrin) (Figure 2H). It is noteworthy that the orientation and distance of PheCE4 from HSP do not suggest any sort of π stabilization. This HSP orientation may explain, in part, the significant reduction in distance between the heme iron and HisF8 N_ε_ (Figure 2D) following αHisE7 protonation, due to weak electrostatic repulsion pushing the heme iron below the porphyrin plane toward HisF8. The implication of HSP in bovine DHb being oriented toward the heme iron more than the HSP of trout IV DHb provides insight regarding the capacity for trout IV DHb to oxidize lipids. The greater tendency of HSP to be in the up position in the fish DHb provides a channel for distal water exit, thus leaving the ferrous heme iron in an unstable, oxidant-accessible, 5-coordinate state.

### HisE7 Protonation Causes the HisF8 N_ε_–Heme Iron Distance (Fe–L_1_) to Decrease

The Fe–L_1_ distance ranges from ∼1.9–2.3 Å for a variety of heme proteins (Hb and Mb) and heme-containing constructs.^43^ Our results display a significant reduction in Fe–L_1_ distance following protonation of HisE7 for both bovine and trout IV DHb (Figure 2D,G, full data set can be seen in Figure S2A,B) compared to their deprotonated states. Although statistically significant, the biological relevance of a decrease in the Fe–L_1_ distance of ∼0.002 nm following distal histidine protonation is unknown. This minute change is complimented by an insignificant change in iron displacement from the heme plane following protonation of the distal histidine in bovine and trout DHb (Figure S2C–E). What is more intriguing is that our data suggests that trout IV DHb has a longer Fe–L_1_ distance (Figure 2D,G) and a more pronounced iron displacement from the heme plane (Figure S2E) compared to bovine DHb, regardless of the HisE7 protonation state. This extended length of the Fe–L_1_ distance and displaced heme iron in trout IV DHb are likely to play an important role in the decreased oxygen affinity. Capece and others^44^ employed QM–MM and found that increasing the Fe–L_1_ distance decreased the oxygen affinity, thus supporting our finding. Interpolating from the Capece data, the Fe–L_1_ distance contributes 316 kcal mol^–1^ nm^–1^ to oxygen binding affinity; thus, the difference in the Fe–L_1_ distance of 0.003 nm between bovine and trout IV accounts for an increase in the binding affinity of 0.86 kcal mol^–1^ favoring the shorter bonded bovine DHb. Further, these researchers indicated that the Fe–L_1_ distance and bond strength were positively correlated with the hydrogen bond strength between H_δ_ (proximal histidine) and neighboring H-bond acceptors. However, when analyzing the distance between N_δ_ (His F8) and the neighboring backbone carbonyl (Leu F4) for trout IV compared to bovine, no difference was observed (data not shown). Although speculative, it may be reasonable to consider that a longer Fe–L_1_ distance may lead to more hemin dissociation following oxidation. In support of this hypothesis, Hargrove and others^45^ created a distal histidine Mb mutant (H64Y) which displayed a 20% longer Fe–L_1_ bond and a ∼20-fold lower hemin affinity compared to the WT Mb. Regarding the iron displacement, the reported difference of ∼0.004 nm between bovine and trout DHb is of relevance. Previous research^46^ has shown that sperm whale Mb (PDB: 1A6N) which displays an increase in iron displacement of 0.006 nm compared to soy legHb (PDB: 1GDJ) also showed a 12× decrease in oxygen affinity. To further support the relevance of the Fe–L_1_ and iron displacement differences between bovine and trout IV, a comparison of a low O_2_ affinity, T-state, DHb (PDB: 1HGA)^47^ and a high O_2_ affinity, R-state, DHb (PDB: 1IBE)^48^ was performed. The α-chain iron displacement was 0.005 nm higher, and the Fe–L_1_ distance was 0.002 nm longer in the low-affinity structure.

### α_1_–β_2_ Interfacial Contact Distances Differ between Species

The interfacial contacts play an important role in the global Hb structure and the tetramer to subunit transition. The tetramer to subunit transition is of biological relevance as Hb dimers present much higher rates of hemin dissociation, particularly in the β-chains.^49^ Samuel and Case^12^ identified 4 sites at the α_1_–β_2_ interface of human HbA which were disrupted following the breakage of the HisF8–iron bond. Although we did not produce any breakage of the HisF8–iron bond, we examined how the interfacial dynamics changed over time when comparing bovine DHb (Figure 3) to trout IV DHb (Figure 4). For bovine DHb, the interfacial distances for all 4 sites were ∼5–6 Å, when averaged across all replicates, for the duration of the simulations (Figure 3). Trout IV DHb only displays 3 interface sites (Figure 5A) due to a substitution of Thr α_1_C6 in bovine for an Ala in trout IV, removing the H-bonding interaction with Arg β_2_C6. Interface sites 1 and 3 of trout IV show distance patterns similar to those of bovine (Figure 4). In fact, when comparing the maximum recorded interfacial distance, no significant difference was found between trout IV and bovine DHb (Figure 5B,D). Further, protonation of HisE7 had no effect on the maximum interfacial distances at sites 1–3 for both trout IV and bovine DHb (Figure 5B–D). In contrast, stark differences were observed at interface site 2 when comparing bovine to trout IV DHb, with trout IV displaying far more variability. Regardless of the HisE7 protonation state, numerous simulations reached a maximum distance of >10 Å at site 2 (Figure 4, middle column), caused by the guanidinium group of Arg β_2_C6 swinging out toward the solvent. Although no significant difference was observed when the maximum distances at site 2 between trout IV and bovine with HisE7 HSD were compared, significance was reached for this comparison when HisE7 was protonated (HSP) (Figure 5C). The site 2 fluctuations observed for trout IV Hb may in part be due to the substitution of Thr (bovine) α_1_C6 for Ala. This replacement for the hydrophobic residue eliminates the hydrogen bond between Thr-OH and Arg-guanidinium, thus increasing the mobility of the guanidinium group and causing the Argβ_2_C6 position to fluctuate. These data suggest that the large changes at site 2 are transient (Figure 4) but may still provide clues as to why trout IV and bovine DHb behave differently at post-mortem pH. To further understand how residue motion influences the interfacial structure, we performed a local, backbone RMSD analysis of the 6 interfacial residues for all models (Figure 5E). No clear backbone transition was found for any of the models, but data seemed to be trending more in an upward direction for trout IV compared to bovine DHb, particularly for the native models (Figure 5E).

**Figure 3:**
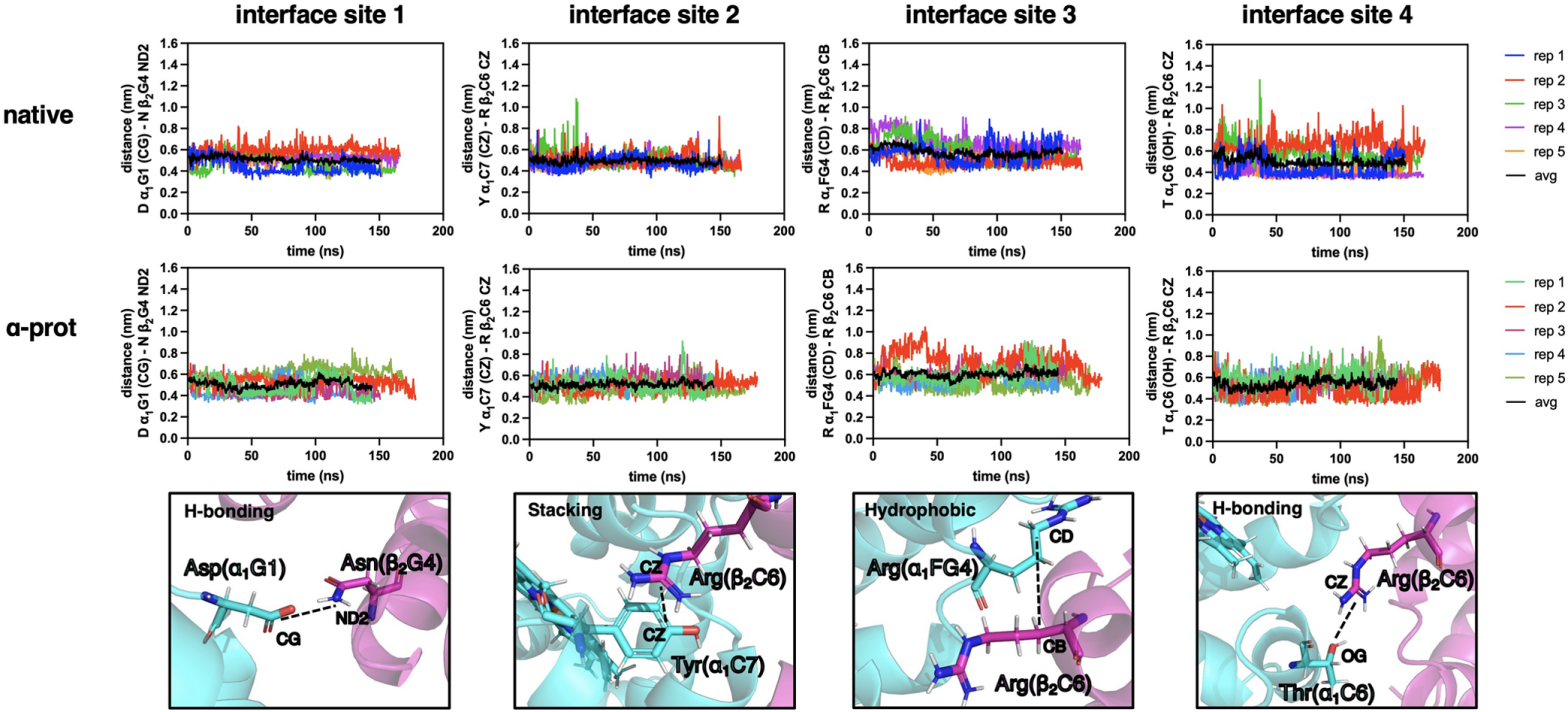
Distance fluctuations at the α_1_β_2_ interface of bovine deoxyhemoglobin (DHb). (Top row) Native bovine DHb. (Middle row) Bovine DHb with protonated HisE7 for both α chains. (Bottom row) Illustration of residues and atoms used to measure the α_1_β_2_ interface distance. Each column corresponds to 1 of the 4 selected interface sites.

**Figure 4:**
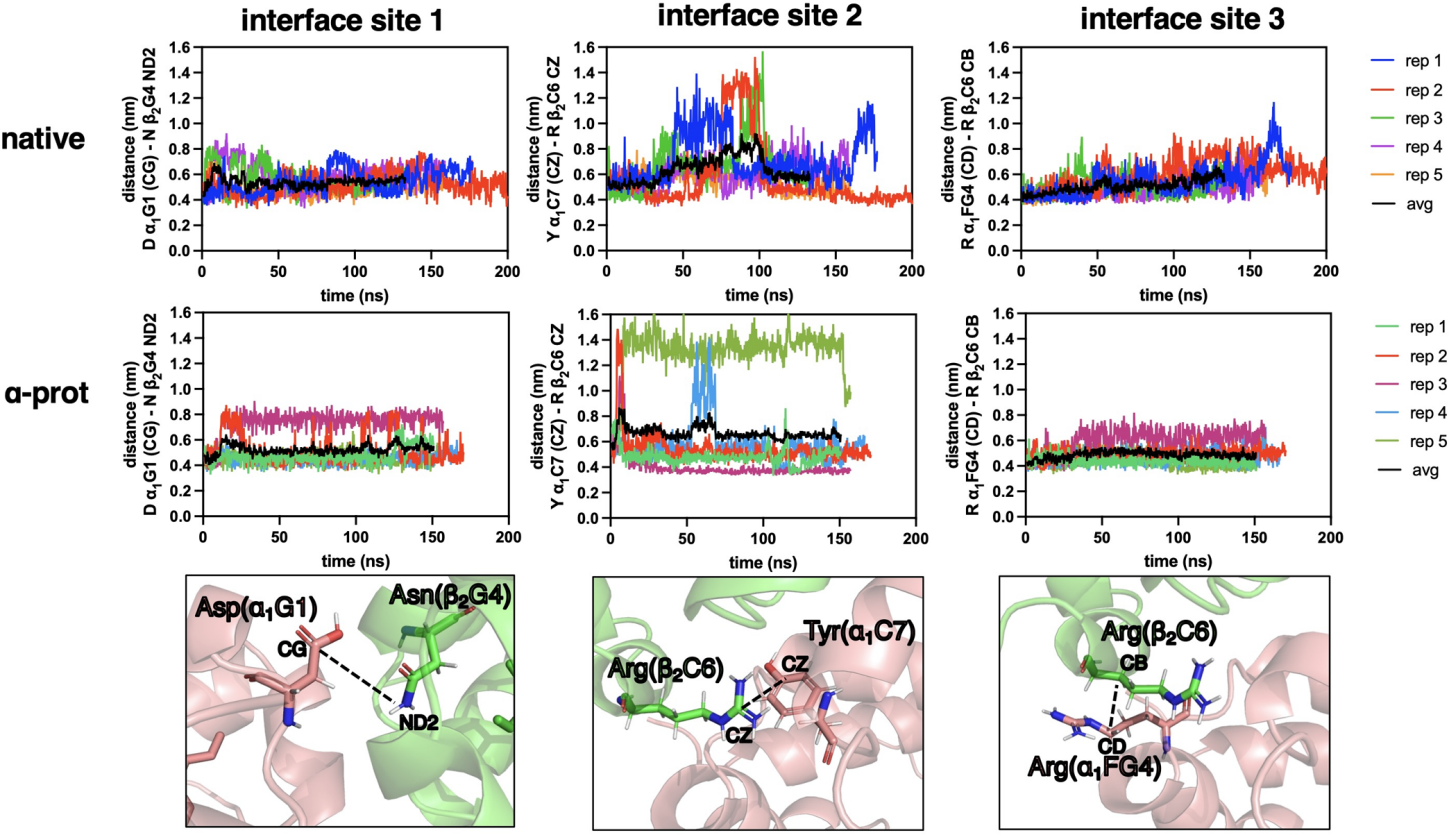
Distance fluctuations at the α_1_β_2_ interface of trout IV deoxyhemoglobin (DHb). (Top row) Native trout IV DHb, (middle row) trout IV DHb with protonated HisE7 for both α chains, and (bottom row) illustration of residues and atoms used to measure the α_1_β_2_ interface distance. Each column corresponds to 1 of the 3 selected interface sites.

**Figure 5:**
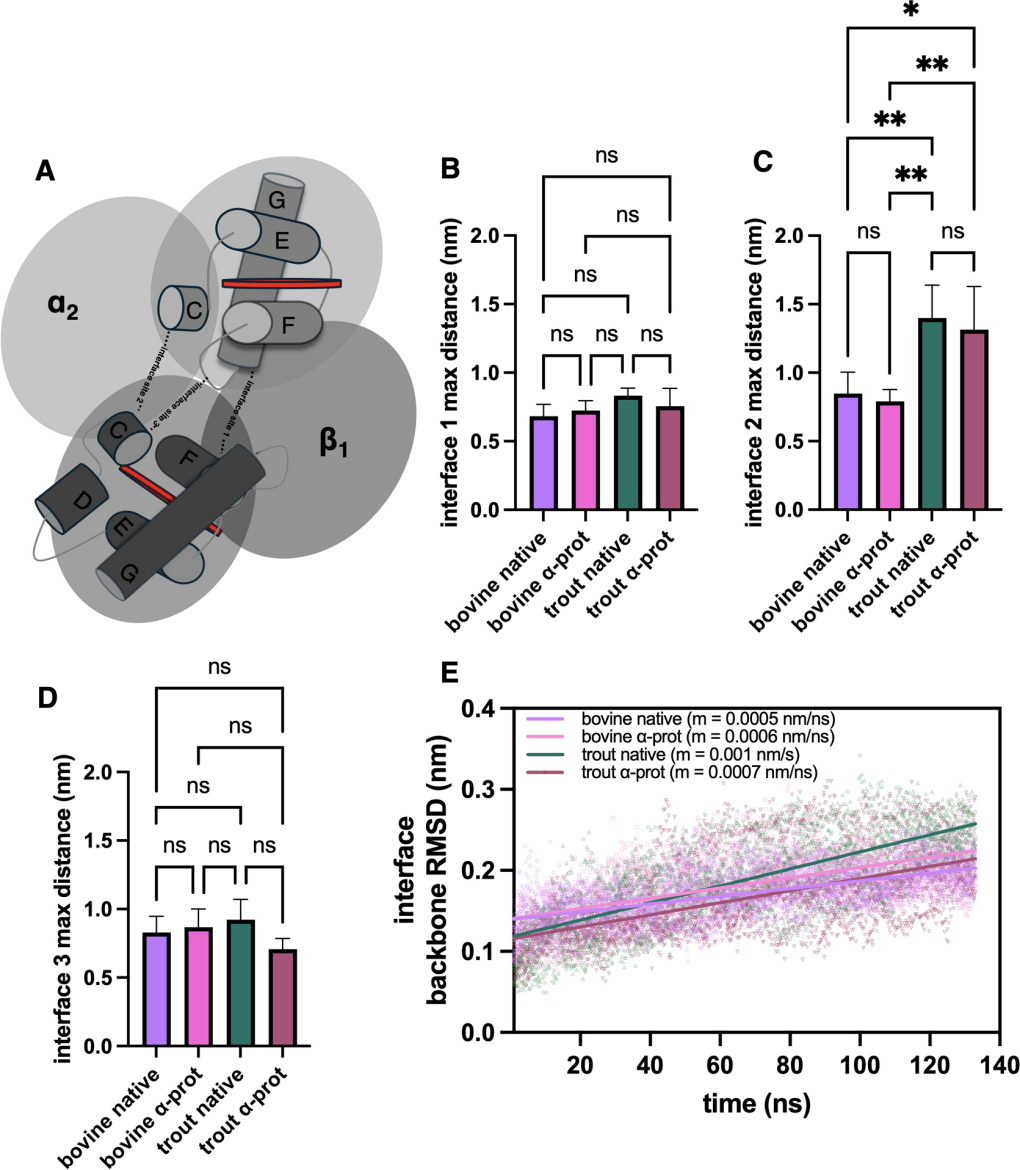
Interspecies comparison of the α_1_β_2_ interface. (A) Schematic of three critical interfacial sites between the G helices (interface site 1), C helices (interface site 2), and α_1_FG–β_2_C (interface site 3). Other helices (i.e., A, B, and H) were removed for clarity. Red disks represent the heme moiety. (B) Interface site 1 maximum recorded distance. (C) Interface site 2 maximum recorded distance. (D) Interface site 3 maximum recorded distance. (E) Local backbone RMSD analysis of 6 interfacial residues bovine and trout IV DHb in their native and α-prot state. Interfacial residues were α_1_C6, α_1_C7, α_1_FG4, α_1_G1, β_2_C6, and β_2_G4. Data were fit to a simple linear regression. Value in parentheses are the rate of change (slope) calculated by the linear regression.

### SEC Reveals Differing Particle Size Distributions for Trout IV and Bovine DHb

To complement our computational findings, we performed SEC to see whether the global architecture of DHb in solution was different between trout IV and bovine. Our results indicate a broader distribution of DHb particles in trout IV compared to bovine (Figure 6), indicating a higher heterogeneity for trout IV DHb. The larger particles are likely aggregates (Figure 6, zone 1), while the smaller particles are likely subunits (Figure 6, zone 3). These data are supported by the unpublished findings of Kristinsson,^50^ who found trout Hb to be largely monomeric at low pH independent of Hb concentration. Conversely, these data contrast with previous results indicating a larger tetramer–dimer dissociation constant for mammalian Hb compared to trout IV;^51,52^ however, in both cases, experimental conditions were different (e.g., higher pH and O_2_ ligated Hb). The relevance of this finding lies in previous research indicating Hb subunits display an increased affinity for lipid membranes^53,54^ and display increased hemin dissociation^49^ compared to Hb tetramers. The elevated membrane interaction would be further exacerbated by the increased hydrophobic area of trout IV Hb compared to bovine (Figure S3). The increased monomeric population of trout IV DHb at low pH may be driven by the labile nature of α_1_–β_2_ as shown by our interfacial analysis from MD simulations (Figure 5).

**Figure 6:**
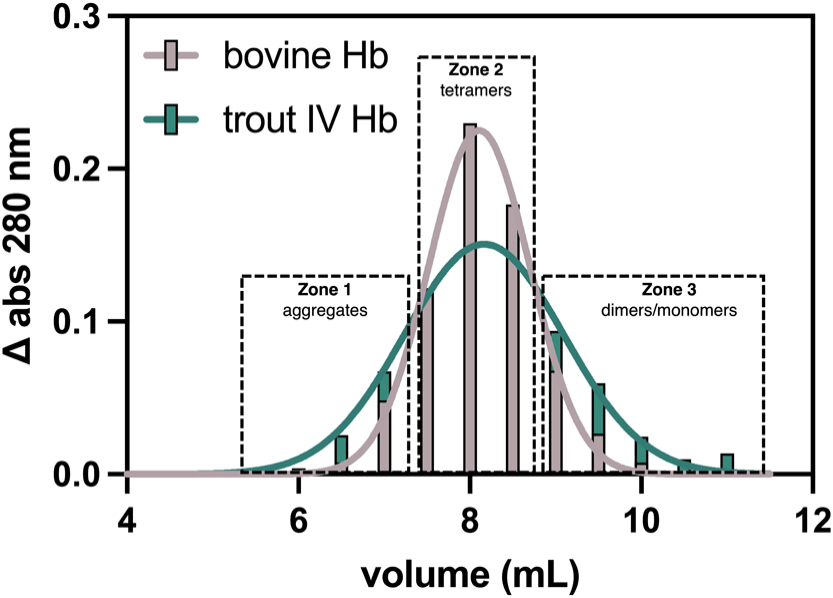
SEC elution profile of trout IV and bovine deoxyhemoglobin at pH 5.7. Separations were performed using Sepharose CL-6B resin with 10 mM MES pH 5.7 as the mobile phase, under the force of gravity. Hemoglobin concentration was 40 μM (heme basis). All buffers were deoxygenated with Ar_(g)_ prior to use, and all column preparation steps along with the separation were performed under a continuous flow of Ar_(g)_. Separations were performed 3 times for each Hb.

### Computed p*K*_a_ of HisE7 Is Different between Fish and Mammals

The electrostatic environment of histidine residues impacts their p*K*_a_ values, which, in turn, affects protein structure/function and histidine-mediated catalysis. Using a Poisson–Boltzmann-based p*K*_a_ calculator,^30^ we calculated the p*K*_a_ of the α-chain distal histidine of Hb from different species (Figure 7). We have included perch among the main species under study because it, like trout IV, displays a strong Root effect.

**Figure 7:**
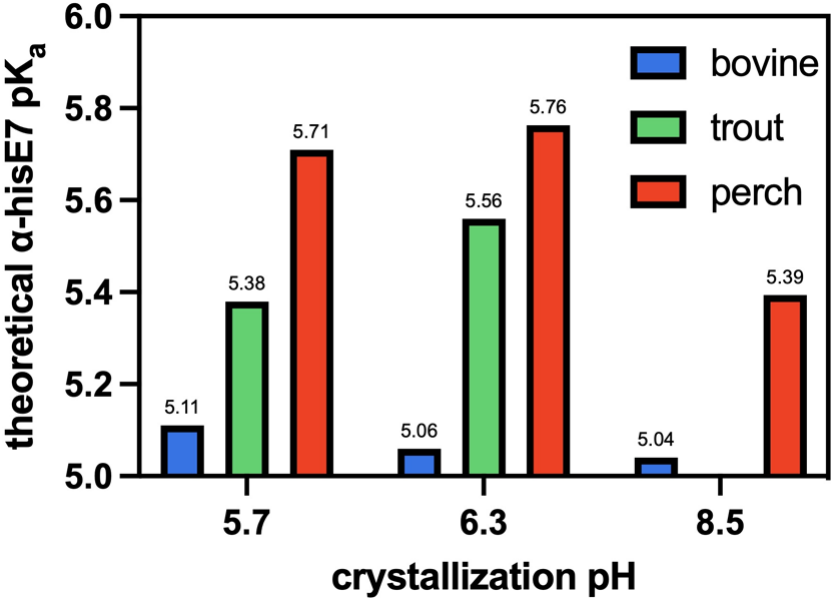
Calculated p*K*_a_ values of α-chain distal histidine E7 (HisE7). p*K*_a_ values of HisE7 on the α chain of hemoglobin crystal structures for bovine [PDB ID: 2qsp (pH 5.7), 2pss (pH 6.3), and 1g0a (pH 8.5)], trout [PDB ID: 3bom (pH 5.7) and 2r1h (pH 6.3)], and perch [PDB ID: 3bj1 (pH 5.7), 3bj2 (pH 6.3), and 3bj3 (pH 8.5)]. p*K*_a_ calculations were performed using PypKa (pH range: 0–12, pH step: 0.25, protein dielectric: 15, solvent dielectric: 80, and ionic strength: 0.1). PypKa does not account for the charges associated with the heme moiety.

Interestingly, we found a trend of αHisE7 p*K*_a_ for perch > trout IV > bovine using crystal structures from pH 5.7 and 6.3 (Figure 7). It should be noted that the differences reported in Figure 7 may fall within the error of PypKa^30^ calculations (RMSE = 0.8); however, the calculated trends are complimented by experimental data. The calculated trend aligns nicely with experimental data presented by Berenbrink et al.,^55^ which reported that the Root effect, a HisE7-dependent phenomenon, was on the order of perch > rainbow trout[Fn fn5] > human hemolysate. Further, Aranda et al.^6^ showed the same trend at pH 5.7 and 6.3 for two other HisE7-dependent phenomenon–hemin loss rates and auto-oxidation. To our knowledge, no direct measure of the distal histidine p*K*_a_ for bovine, trout IV, and perch Hb has been reported. The elevated p*K*_a_ values of the fish hemoglobin may be due to a combination of the following factors (i) charged LysE10 (Thr in trout) in bovine favoring the neutral form of HisE7,^56^ (ii) HisE7 proximity to the negatively charged heme propionate,^57^ and (iii) TrpCE4 (Phe in bovine) which would stabilize HSP.^40,58^ Unfortunately, using our current data analysis methods, we are unable to calculate the p*K*_a_ of HisE7 throughout the duration of the MD simulations. Future work should explore the implementation of a postprocessing method to calculate this value as the position of HisE7 changes or perform constant pH MD simulations^59^ to more reasonably simulate the HisE7 protonation state.

### WCE4F In Silico Mutation in Trout Did Not Keep the Distal Histidine Down

Whether αHisE7 is in the up or down position, adjacent aromatic residues influence its mobility and position.^39^ We identified position CE4 as a heme pocket residue with different identities between bovine (Phe) and trout IV (Trp) DHb. Figure 1A shows that protonated αHisE7 stays in the down position more often (Figure 2B) compared to when αHisE7 was deprotonated (Figure 1B) in bovine DHb. To examine how PheCE4 impacts the αHisE7 position, we performed an in silico mutation in trout IV Hb αTrpCE4 → Phe (Figure S4A). If the identity of residue CE4 determined the position αHisE7, we would expect that following protonation, the swing up occurrences would decrease, and the time to swing up would increase (Figure 2B–D). In contrast, following protonation, our data show that the Phe mutation leads to a marginal increase in normalized swing out occurrences from 7 to 7.6 (Figure S4B) and a decrease in the swing up time (Figure S4C). These data indicate that the αHisE7 position is not solely determined by the identity of residue CE4. This does not support our initial hypothesis that TrpCE4 determines the ability of HisE7 HSP to readily swing out. Despite CE4 mutation not incurring effects on the HisE7 orientation in trout IV DHb, there may be downstream effects on the local and global structure; however, we did not perform any formal analysis to assess this.

### Bovine DHb Displays Increased Nitrite Reductase Activity at Low pH

By using SASA for residue E11 as a proxy for solvent access to the heme pocket, we found that solvent access decreased upon HisE7 protonation in bovine (Figure 8A) and increased (Figure 8B) in trout IV DHb. We were unable to make a direct comparison between trout and bovine because of the differences in the identity of residue E11 (bovine = Val and trout IV = Ile). We also measured the SASA of the heme moiety itself which allows for direct species comparison and found that in the native form, the heme SASA was significantly higher in trout IV compared to bovine (Figure 8C) and interestingly no significant differences following protonation of HisE7. Due to the stabilization of the trout IV αHSP-E7 in the up position (bovine αHSP-E7 stays pointed down toward the heme), a channel for access of small molecules to the heme iron is formed; conversely, the stabilization of bovine αHSP-E7 in the down position may lead to alternate nitrite channels (Figure 9A,B). To corroborate this experimentally, we evaluated each of the Hb’s for their nitrite reductase activity at low (5.7) and physiological (7.4) pH, anticipating a reduced nitrite reductase activity for bovine DHb, particularly at low pH due to heme cavity obstruction.

**Figure 8:**
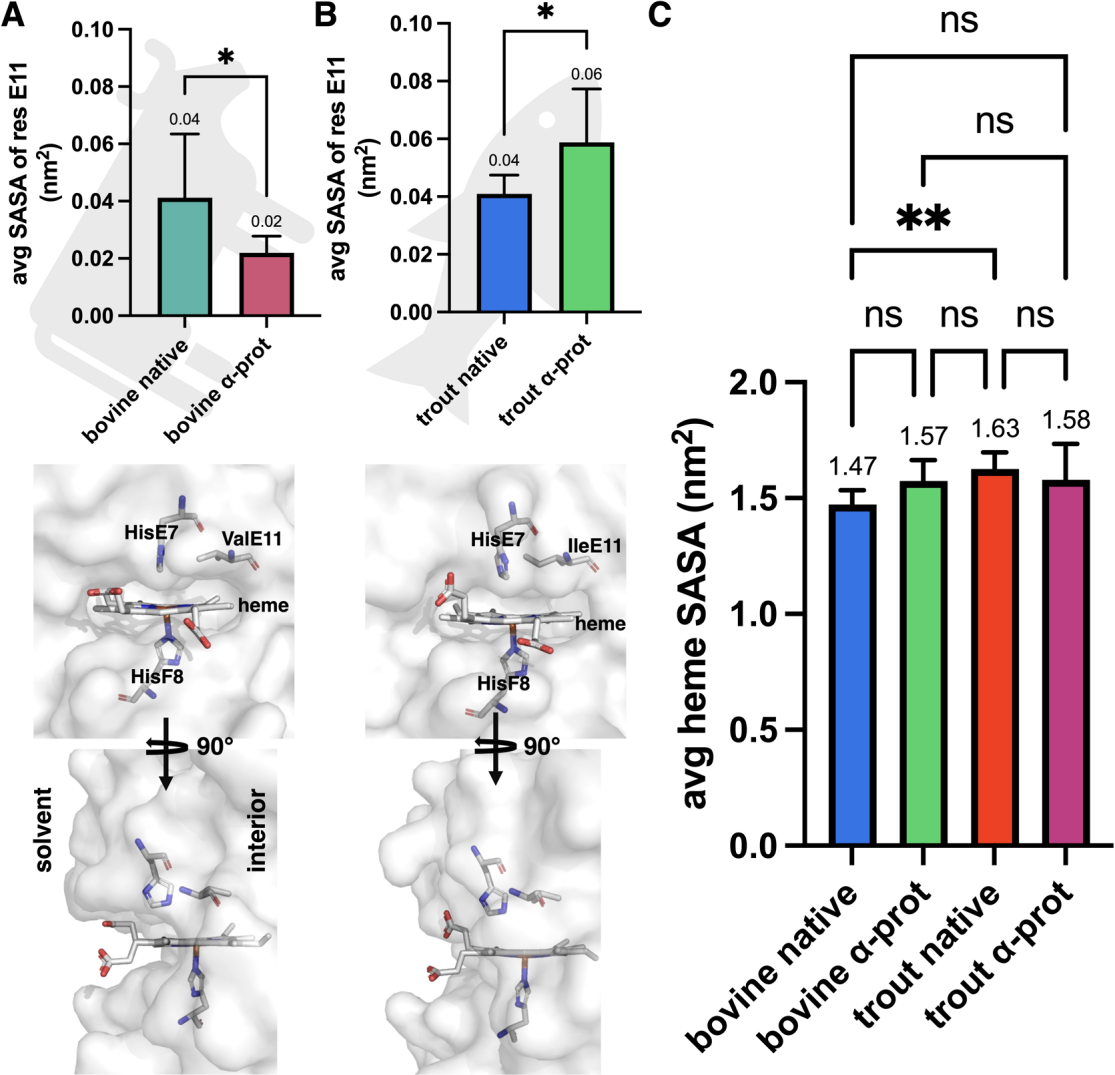
SASA of residue E11 in bovine (A) and trout IV DHb (B). SASA of heme moiety for bovine and trout IV DHb (C). (A–C) Data are presented as an average SASA for residue E11/heme from the α chain, equating to 10 data points per group. Averages were calculated from 1 to 133 ns for all treatments and all groups.

**Figure 9:**
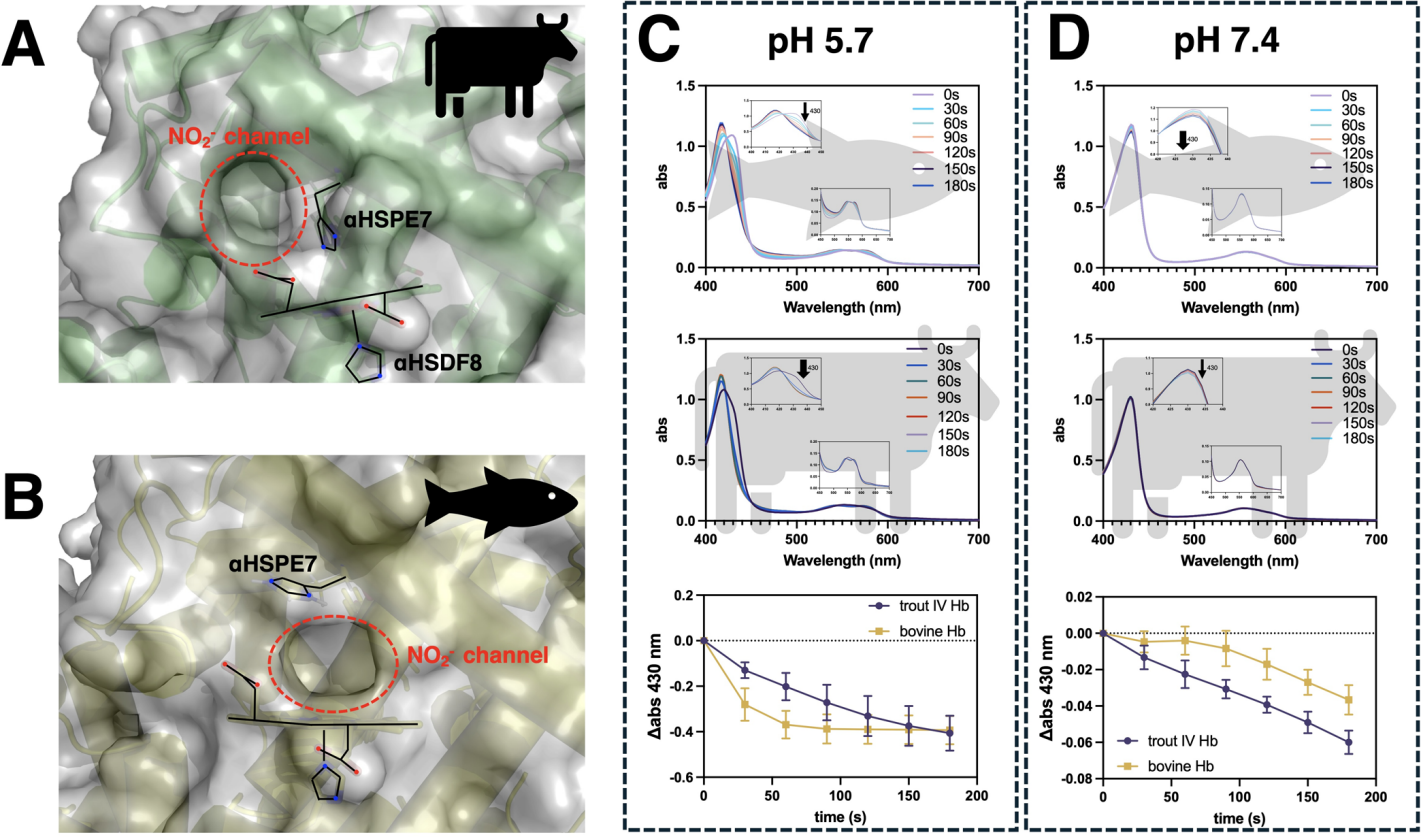
Hemoglobin nitrite reductase activity at pH 5.7 and pH 7.4. (A) Possible nitrite access channel to the bovine heme pocket sampled from MD simulations. (B) Possible nitrite access channel to trout IV heme pocket samples from MD simulations. (C) Electronic absorption spectra of trout IV (top) and bovine (middle) during anaerobic reaction with NaNO_2_ in 10 mM MES pH 5.7. (bottom) Nitrite reductase activity of select Hb at pH 5.7 as measured by the change in abs at 430 nm. (D) Electronic absorption spectra of trout IV (top) and bovine (middle) during anaerobic reaction with NaNO_2_ in 10 mM sodium phosphate pH 7.4. (bottom) Nitrite reductase activity of select Hb at pH 7.4 as measured by the change in abs at 430 nm. (C,D) All reactions were performed at room temperature (22 °C) with ∼10 μM Hb, 19.2 μg glucose oxidase/mL, 1.9 μg catalase/mL, excess sodium dithionite_(s)_, in 0.3% d-glucose 10 mM MES pH 5.7, or 10 mM sodium phosphate buffer pH 7.4. NaNO_2_ was added at 1.35 mM. All buffers and stock solutions were bubbled with Ar_(g)_ to ensure anaerobic conditions.

Our system contained DHb with excess sodium dithionite (DT), so the ferric heme produced during the catalytic cycle gets reduced to ferrous heme by the excess DT, then will bind NO, producing predominantly ferrous nitrosyl heme with small amounts of ferric heme.^35^ In contrast to our hypothesis, bovine DHb displayed greater nitrite reductase activity at pH 5.7 (Figure 9C), as measured by a decrease in the absorbance at 430 nm. This may indicate that the ability of αHSP-E7 to stay pointed down toward the heme may be more important than a more-open heme cavity. This notion is strengthened by the work of Martí and others,^60^ who showed that the nitrite affinity in the catalytic site of nitrite reductase from *Pseudomonas aeruginosa* increased by an order of magnitude when one of the catalytic histidine’s was protonated. This sheds light on the importance of αHSP-E7 proximity to the heme iron during the nitrite reductase catalytic cycle of DHb.^61^ Expectedly, when evaluating the nitrite reductase activity at pH 7.4, the reaction proceeded much slower,^62^ with trout IV displaying a faster rate of nitrite reduction (Figure 9D). This finding agrees with a previous report indicating a faster rate of nitrite reduction for rainbow trout hemolysate compared to mammalian hemolysate at physiological pH.^63^ This could be explained by the elevated computed p*K*_a_ of αHisE7 of fish Hb compared to bovine (Figure 7), which would lead to a larger percentage of protonated αHisE7, for effective nitrite reduction.^60,61^

It has been well studied that Hb from fish vs mammalian possesses diverging redox properties (*k*_ox_)^6^ and hemin dissociation,^6,7^ both of which contribute to their pro-oxidant properties in muscle tissues. However, deep mechanistic insights are still lacking. Here, we present dynamic, atomic-level analysis of Hb α-distal histidine in various protonation states and interfacial analysis to understand why DHb from fish and mammals possess unique functional attributes. Our research led us to the following key findings: (i) following protonation of bovine Hb αHisE7, it tends to stay pointed toward the heme. (ii) Heme pocket parameters (swing up events, swing up speed, and Fe–L_1_ distance) are different between bovine and trout IV Hb. (iii) α_1_–β_2_ interfacial differences may destabilize trout IV Hb. (iv) Bovine DHb possesses a higher nitrite reductase activity at low pH. (v) Trout IV DHb possesses a more heterogeneous particle size distribution compared to bovine DHb at low pH. Taken together, these findings lead us to the following pro-oxidative pathway of fish Hb: At low pH, there is increased solvent access to the pro-oxidant heme and a weak tetramer interface, causing hydrophobic monomers and dimers to interact and oxidize lipid membranes.

Although we have proposed some compelling data to identify key interactions within the heme pocket of bovine and trout IV DHb, several limitations exist. The first being the limitations of additive force fields (e.g., CHARMM36m) to describe cation–π interactions,^64^ which are particularly important when examining the interaction of positively charged distal histidine (cation) with aromatic residues (π-system). Attempts have been made to improve the accuracy of cation–π interactions by optimizing atom pair-specific Lennard-Jones parameters (Drude polarizable force field)^64,65^ and through the introduction of a distance (1/*r* ^4^) term when calculating the nonbonded potential energy between cation and aromatic atoms.^66^ Drude force fields have been implemented into the CHARMM-GUI^65^ input generator suite as *Drude prepper*; however, residues are assigned as protein, DNA, RNA, water, or carbohydrate, thus providing no classification for heme moieties (Figure S5). Second, no QM optimizations were performed for the heme moiety. Our model (based on heme parameters) was ferrous DHb with water as the sixth ligand, which saw the water leave the heme pocket in some simulations, which would cause the ferrous iron charge to increase. Future works could implement QM–MM to study critical heme pocket conformations and evaluate the Hb’s in their ferric (met) form. Finally, to more quantitively describe the HisE7 motion, extended simulations (>200 ns) combined with advanced sampling techniques should be employed, for example, a Markov state model.^67^

## Supplementary Material



## Data Availability

All models and trajectory files for all simulation replicates can be downloaded from the ScienceDB repository (10.57760/sciencedb.19468). This work is licensed under CC BY-NC 4.0. To view a copy of this license, visit https://creativecommons.org/licenses/by-nc/4.0/.

## Footnotes

a The RMSD between the crystal structure for bovine COHb (2qss) and bovine metHb (2qsp) is 0.11, indicating minimal global structural differences between metHb and COHb.

b Best results were achieved using solid sodium dithionite. Sodium dithionite in solution decays rapidly.

c Retention time (RT) alignment was performed to account for retention time differences due to slight variation in column packing. The peak shape for each of the DHb tested was the same for all experiments, despite minor differences in RT. The RT peak shift ranged from 0 to 1.5 mL (avg = 0.75) across all replicates.

d
^“^Native” refers to constructs where the distal HisE7 is in the HSD form. “Prot” refers to constructs where the distal HisE7 is in the HSP form. See Figures 1A and S1 for visualization.

e This group used hemolysate which would contain trout I–IV. Trout I–III have a less pronounced Root effect than trout IV, so it is possible that the Root effect of perch and trout IV is approximately equal. Trout IV accounts for ∼65% of Hb in trout hemolysate.

## Notes

GROMACS minimization input; GROMACS equilibration input; GROMACS production input parameters for trout IV and bovine (native/α-prot); GROMACS production input parameters for trout IV DHb WCE4F; protonated sites on bovine DHb; protonated sites on trout IV DHb; MD simulation lengths; bovine DHb atom numbers for post-processing analysis; trout IV DHb atom numbers for post-processing analysis; histidine naming conventions and basic simulation parameters; distance between HisF8 N_ε_ and heme iron (Fe–L_1_); trout IV and bovine Hb hydrophobic area; trout IV W46F mutant HisE7 movement; failure of *Drude prepper* to categorize HEME moiety; orientation of π–π stacking interactions; and CHARMM36 additive potential energy function (PDF)

## Abbreviations

Hb: hemoglobin
DHb: deoxyhemoglobin
HSP: protonated histidine
HSE: neutral histidine (N_ε_H)
HSD: neutral histidine (N_δ_H)
HisE7: hemoglobin distal histidine
HisF8: hemoglobin proximal histidine
Fe–L_1_: distance between heme iron and HisF8 nitrogen
TBARS: thiobarbituric acid reactive substances
MDA: malondialdehyde
HHE: 4-hydroxy-2-hexenal
MD: molecular dynamics
SEC: size exclusion chromatography
