# Supplementary material for: Molecular Dynamics Reveals Unique Distal Histidine Positions and Interfacial Transitions of Fish and Mammalian Deoxyhemoglobin at Post-mortem pH
Source: J Agric Food Chem. 2025 Aug 8;73(33):21059–71. doi: 10.1021/acs.jafc.5c05805 (PMC12503364; doi:10.1021/acs.jafc.5c05805)
Supplement: Supplementary file 1 [file jf5c05805_si_001.pdf]

Supporting Information

Molecular Dynamics Reveal Unique Distal Histidine Positions and Interfacial Transitions of Fish and Mammalian Deoxyhemoglobin at Post-Mortem pH.

Sean Baker[a] and Mark P. Richards[a,b*]

[a] Department of Food Science, University of Wisconsin-Madison, Madison, WI 53706, USA

[b] Department of Animal and Dairy Sciences, University of Wisconsin-Madison, Madison, WI 53706, USA

*Corresponding author. Tel.: +1 507-481-7384

**Email Address:* sbaker8@wisc.edu

GROMACS minimization input
GROMACS equilibration input
GROMACS production input parameters for trout IV and bovine DHb (native/α-prot)
GROMACS production input parameters for trout IV DHb WCE4F
Table S1. Protonated sites on bovine DHb.
Table S2. Protonated sites on trout IV DHb.
Table S3. All simulation lengths.
Table S4. Bovine DHb atom numbers for post processing analysis.
Table S5. Trout IV DHb atom numbers for post processing analysis.
Figure S1. Histidine naming conventions and simulation RMSD, pressure and volume.
Figure S2. Distance between HisF8 N_ε and heme iron (Fe-L_1).
Figure S3. Trout IV and bovine Hb hydrophobic area.
Figure S4. Trout IV W46F mutant HisE7 movement.
Figure S5. Failure of *Drude prepper* to categorize HEME moiety.
Figure S6. Orientation of π-π stacking interactions.
Equation S1-3. CHARMM36 additive potential energy function.
Supporting References

GROMACS minimization input

```
integrator              = steep
emtol                   = 1000.0
nsteps                  = 5000
nstlist                 = 10
cutoff-scheme           = Verlet
rlist                   = 1.2
vdwtype                 = Cut-off
vdw-modifier            = Force-switch
rvdw_switch             = 1.0
rvdw                    = 1.2
coulombtype             = PME
rcoulomb                = 1.2
;
constraints             = h-bonds
constraint_algorithm    = LINCS
```

GROMACS equilibration input

```
integrator              = md
dt                      = 0.001
nsteps                  = 125000
nstxout-compressed      = 5000
nstxout                 = 0
nstvout                 = 0
nstfout                 = 0
nstcalcenergy           = 100
nstenergy               = 1000
nstlog                  = 1000
;
cutoff-scheme           = Verlet
nstlist                 = 20
rlist                   = 1.2
vdwtype                 = Cut-off
vdw-modifier            = Force-switch
rvdw_switch             = 1.0
rvdw                    = 1.2
coulombtype             = PME
rcoulomb                = 1.2
;
tcoupl                  = v-rescale
```

```
tc_grps                 = SOLU SOLV
tau_t                   = 1.0 1.0
ref_t                   = 298 298
;
constraints             = h-bonds
constraint_algorithm    = LINCS
;
nstcomm                 = 100
comm_mode               = linear
comm_grps               = SOLU SOLV
;
gen-vel                 = yes
gen-temp                = 298
gen-seed                = -1
```

GROMACS production input parameters for trout IV and bovine (native/α-prot)

```
integrator               = md
tinit                    = 0
dt                       = 0.004
nsteps                   = 50000000
init-step                = 0
simulation-part          = 1
mts                      = false
mass-repartition-factor  = 1
comm-mode                = Linear
nstcomm                  = 100
bd-fric                  = 0
ld-seed                  = -268730625
emtol                    = 10
emstep                   = 0.01
niter                    = 20
fcstep                   = 0
nstcgsteep               = 1000
nbfgscorr                = 10
rtpi                     = 0.05
nstxout                  = 0
nstvout                  = 50000
nstfout                  = 50000
nstlog                   = 1000
nstcalcenergy            = 100
nstenergy                = 1000
nstxout-compressed       = 50000
compressed-x-precision   = 1000
```

```
cutoff-scheme                  = Verlet
nstlist                        = 20
pbc                            = xyz
periodic-molecules             = false
verlet-buffer-tolerance        = 0.005
verlet-buffer-pressure-tolerance = -1
rlist                          = 1.27
coulombtype                    = PME
coulomb-modifier               = Potential-shift
rcoulomb-switch                = 0
rcoulomb                       = 1.2
epsilon-r                      = 1
epsilon-rf                     = inf
vdw-type                       = Cut-off
vdw-modifier                   = Force-switch
rvdw-switch                    = 1
rvdw                           = 1.2
DispCorr                       = No
table-extension                = 1
fourierspacing                 = 0.12
fourier-nx                     = 84
fourier-ny                     = 84
fourier-nz                     = 84
pme-order                      = 4
ewald-rtol                     = 1e-05
ewald-rtol-lj                  = 0.001
lj-pme-comb-rule               = Geometric
ewald-geometry                 = 3d
epsilon-surface                = 0
ensemble-temperature-setting   = constant
ensemble-temperature           = 298
tcoupl                         = Nose-Hoover
nsttcouple                     = 10
nh-chain-length                = 1
print-nose-hoover-chain-variables = false
pcoupl                         = Parrinello-Rahman
pcoupltype                     = Isotropic
nstpcouple                     = 50
tau-p                          = 5
compressibility (3x3):
   compressibility[    0]={ 4.50000e-05,  0.00000e+00,  0.00000e+00}
   compressibility[    1]={ 0.00000e+00,  4.50000e-05,  0.00000e+00}
   compressibility[    2]={ 0.00000e+00,  0.00000e+00,  4.50000e-05}
ref-p (3x3):
```

```
    ref-p[    0]={ 1.00000e+00,  0.00000e+00,  0.00000e+00}
    ref-p[    1]={ 0.00000e+00,  1.00000e+00,  0.00000e+00}
    ref-p[    2]={ 0.00000e+00,  0.00000e+00,  1.00000e+00}
  refcoord-scaling               = No
  posres-com (3):
    posres-com[0]= 0.00000e+00
    posres-com[1]= 0.00000e+00
    posres-com[2]= 0.00000e+00
  posres-comB (3):
    posres-comB[0]= 0.00000e+00
    posres-comB[1]= 0.00000e+00
    posres-comB[2]= 0.00000e+00
  QMMM                           = false
qm-opts:
  ngQM                           = 0
  constraint-algorithm           = Lincs
  continuation                   = false
  Shake-SOR                      = false
  shake-tol                      = 0.0001
  lincs-order                    = 4
  lincs-iter                     = 1
  lincs-warnangle                = 30
  nwall                          = 0
  wall-type                      = 9-3
  wall-r-linpot                  = -1
  wall-atomtype[0]               = -1
  wall-atomtype[1]               = -1
  wall-density[0]                = 0
  wall-density[1]                = 0
  wall-ewald-zfac                = 3
  pull                           = false
  awh                            = false
  rotation                       = false
  interactiveMD                  = false
  disre                          = No
  disre-weighting                = Conservative
  disre-mixed                    = false
  dr-fc                          = 1000
  dr-tau                         = 0
  nstdisreout                    = 100
  orire-fc                       = 0
  orire-tau                      = 0
  nstorireout                    = 100
  free-energy                    = no
```

```
cos-acceleration              = 0
deform (3x3):
   deform[    0]={ 0.00000e+00,  0.00000e+00,  0.00000e+00}
   deform[    1]={ 0.00000e+00,  0.00000e+00,  0.00000e+00}
   deform[    2]={ 0.00000e+00,  0.00000e+00,  0.00000e+00}
simulated-tempering           = false
swapcoords                    = no
userint1                      = 0
userint2                      = 0
userint3                      = 0
userint4                      = 0
userreal1                     = 0
userreal2                     = 0
userreal3                     = 0
userreal4                     = 0
applied-forces:
  electric-field:
    x:
      E0                      = 0
      omega                   = 0
      t0                      = 0
      sigma                   = 0
    y:
      E0                      = 0
      omega                   = 0
      t0                      = 0
      sigma                   = 0
    z:
      E0                      = 0
      omega                   = 0
      t0                      = 0
      sigma                   = 0
  density-guided-simulation:
    active                    = false
    group                     = protein
    similarity-measure        = inner-product
    atom-spreading-weight     = unity
    force-constant            = 1e+09
    gaussian-transform-spreading-width = 0.2
    gaussian-transform-spreading-range-in-multiples-of-width = 4
    reference-density-filename = reference.mrc
    nst                       = 1
    normalize-densities       = true
    adaptive-force-scaling    = false
```

```
      adaptive-force-scaling-time-constant = 4
      shift-vector               =
      transformation-matrix      =
    qmmm-cp2k:
      active                   = false
      qmgroup                  = System
      qmmethod                 = PBE
      qmfilenames              =
      qmcharge                 = 0
      qmmultiplicity           = 1
grpopts:
   nrdf:      22735      172911
   ref-t:        298        298
   tau-t:          1          1
annealing:          No          No
annealing-npoints:           0           0
   acc:            0           0           0
   nfreeze:           N           N           N
   energygrp-flags[  0]: 0
```

<u>GROMACS production input parameters for trout IV DHb WCE4F</u>

```
   integrator                    = md
   tinit                         = 0
   dt                            = 0.004
   nsteps                        = 50000000
   init-step                     = 0
   simulation-part               = 1
   mts                           = false
   mass-repartition-factor       = 1
   comm-mode                     = Linear
   nstcomm                       = 100
   bd-fric                       = 0
   ld-seed                       = -2100234
   emtol                         = 10
   emstep                        = 0.01
   niter                         = 20
   fcstep                        = 0
   nstcgsteep                    = 1000
   nbfgscorr                     = 10
   rtpi                          = 0.05
   nstxout                       = 0
   nstvout                       = 0
```

```
nstfout                  = 0
nstlog                   = 1000
nstcalcenergy            = 100
nstenergy                = 1000
nstxout-compressed       = 25000
compressed-x-precision   = 1000
cutoff-scheme            = Verlet
nstlist                  = 20
pbc                      = xyz
periodic-molecules       = false
verlet-buffer-tolerance  = 0.005
verlet-buffer-pressure-tolerance = -1
rlist                    = 1.269
coulombtype              = PME
coulomb-modifier         = Potential-shift
rcoulomb-switch          = 0
rcoulomb                 = 1.2
epsilon-r                = 1
epsilon-rf               = inf
vdw-type                 = Cut-off
vdw-modifier             = Force-switch
rvdw-switch              = 1
rvdw                     = 1.2
DispCorr                 = No
table-extension          = 1
fourierspacing           = 0.12
fourier-nx               = 84
fourier-ny               = 84
fourier-nz               = 84
pme-order                = 4
ewald-rtol               = 1e-05
ewald-rtol-lj            = 0.001
lj-pme-comb-rule         = Geometric
ewald-geometry           = 3d
epsilon-surface          = 0
ensemble-temperature-setting   = constant
ensemble-temperature     = 298
tcoupl                   = V-rescale
nsttcouple               = 50
nh-chain-length          = 0
print-nose-hoover-chain-variables = false
pcoupl                   = C-rescale
pcoupltype               = Isotropic
nstpcouple               = 100
```

```
   tau-p                          = 5
   compressibility (3x3):
      compressibility[    0]={ 4.50000e-05,  0.00000e+00,  0.00000e+00}
      compressibility[    1]={ 0.00000e+00,  4.50000e-05,  0.00000e+00}
      compressibility[    2]={ 0.00000e+00,  0.00000e+00,  4.50000e-05}
   ref-p (3x3):
      ref-p[    0]={ 1.00000e+00,  0.00000e+00,  0.00000e+00}
      ref-p[    1]={ 0.00000e+00,  1.00000e+00,  0.00000e+00}
      ref-p[    2]={ 0.00000e+00,  0.00000e+00,  1.00000e+00}
   refcoord-scaling               = No
   posres-com (3):
      posres-com[0]= 0.00000e+00
      posres-com[1]= 0.00000e+00
      posres-com[2]= 0.00000e+00
   posres-comB (3):
      posres-comB[0]= 0.00000e+00
      posres-comB[1]= 0.00000e+00
      posres-comB[2]= 0.00000e+00
   QMMM                           = false
qm-opts:
   ngQM                           = 0
   constraint-algorithm           = Lincs
   continuation                   = true
   Shake-SOR                      = false
   shake-tol                      = 0.0001
   lincs-order                    = 4
   lincs-iter                     = 1
   lincs-warnangle                = 30
   nwall                          = 0
   wall-type                      = 9-3
   wall-r-linpot                  = -1
   wall-atomtype[0]               = -1
   wall-atomtype[1]               = -1
   wall-density[0]                = 0
   wall-density[1]                = 0
   wall-ewald-zfac                = 3
   pull                           = false
   awh                            = false
   rotation                       = false
   interactiveMD                  = false
   disre                          = No
   disre-weighting                = Conservative
   disre-mixed                    = false
   dr-fc                          = 1000
```

```
dr-tau                    = 0
nstdisreout               = 100
orire-fc                  = 0
orire-tau                 = 0
nstorireout               = 100
free-energy               = no
cos-acceleration          = 0
deform (3x3):
   deform[    0]={ 0.00000e+00,  0.00000e+00,  0.00000e+00}
   deform[    1]={ 0.00000e+00,  0.00000e+00,  0.00000e+00}
   deform[    2]={ 0.00000e+00,  0.00000e+00,  0.00000e+00}
simulated-tempering       = false
swapcoords                = no
userint1                  = 0
userint2                  = 0
userint3                  = 0
userint4                  = 0
userreal1                 = 0
userreal2                 = 0
userreal3                 = 0
userreal4                 = 0
applied-forces:
  electric-field:
    x:
      E0                  = 0
      omega               = 0
      t0                  = 0
      sigma               = 0
    y:
      E0                  = 0
      omega               = 0
      t0                  = 0
      sigma               = 0
    z:
      E0                  = 0
      omega               = 0
      t0                  = 0
      sigma               = 0
  density-guided-simulation:
    active                = false
    group                 = protein
    similarity-measure    = inner-product
    atom-spreading-weight = unity
    force-constant        = 1e+09
```

```
      gaussian-transform-spreading-width = 0.2
      gaussian-transform-spreading-range-in-multiples-of-width = 4
      reference-density-filename = reference.mrc
      nst                    = 1
      normalize-densities        = true
      adaptive-force-scaling     = false
      adaptive-force-scaling-time-constant = 4
      shift-vector               =
      transformation-matrix      =
    qmmm-cp2k:
      active                  = false
      qmgroup                 = System
      qmmethod                = PBE
      qmfilenames             =
      qmcharge                = 0
      qmmultiplicity          = 1
grpopts:
   nrdf:     23347     172611
   ref-t:       298        298
   tau-t:          1          1
annealing:          No          No
annealing-npoints:           0           0
   acc:            0           0           0
   nfreeze:           N           N           N
   energygrp-flags[  0]: 0
```

Table S1. Protonated sites on bovine deoxyhemoglobin (PDB ID: 2qsp) pH 5.7. Letters in the parenthesis denote whether the residue is solvated (S), protein buried (P), or intermediate/interface (I). Solvent exposure was calculated using GETAREA by calculating the ratio of side-chain surface area to "random coil" per residue. Residues are solvated if this ratio is >50% (S) and buried if the ratio is <20% (P).

	Bovine							
	native				α-prot			
	**α1**	**β1**	**α2**	**β2**	**α1**	**β1**	**α2**	**β2**
**1**	HSP20 (S)	GLUP21 (S)	HSP20 (S)	ASPP98 (P)	HSP20 (S)	GLUP21 (S)	HSP20 (S)	ASPP98 (P)
**2**	HSP50 (S)		HSP50 (S)		HSP50 (S)		HSP50 (S)	
**3**					HSP58 (I)		HSP58	
**4**	HSP72 (I)		HSP72 (S)		HSP72 (I)		HSP72 (S)	
**5**	HSP112 (I)		HSP112 (I)		HSP112 (I)		HSP112 (I)	

Table S2. Protonated sites on trout IV deoxyhemoglobin (PDB ID: 3bom) pH 5.7. Letters in the parenthesis denote whether the residue is solvated (S), protein buried (P), or intermediate/interface (I). Solvent exposure was calculated using GETAREA by calculating the ratio of side-chain surface area to "random coil" per residue. Residues are solvated if this ratio is >50% (S) and buried if the ratio is <20% (P).

	Trout IV							
	native				α-prot			
	**α1**	**β1**	**α2**	**β2**	**α1**	**β1**	**α2**	**β2**
**1**	ASPP95 (P)	HSP69 (S)	ASPP95 (P)	HSP69 (I)	ASPP95 (P)	HSP69 (S)	ASPP95 (P)	HSP69 (I)
**2**		GLUP129 (I)		GLU129 (I)	HSP59 (I)	GLUP129 (I)	HSP59 (I)	GLU129 (I)

Table S3. MD simulation time for all models.

	Simulation time (ns)					
**Model**	**Rep 1**	**Rep 2**	**Rep 3**	**Rep 4**	**Rep 5**	**Sum**
**Bovine native**	151	166.2	162.4	165.2	152.2	797
**Bovine α-prot**	144.2	178.4	150.6	145.0	168.8	787
**Trout native**	177	200	133	158.8	161.2	830
**Trout α-prot**	150.8	170.6	157	166.4	157.2	802
**Trout WCE4 native**	170.7	141.9	145.7	136.5	165.7	760.5
**Trou WCE4 α-prot**	152.3	133.8	141	145.3	132.2	704.6

Table S4. Bovine DHb atom and residue numbers for post processing analysis.

	Bovine							
	native				α-prot			
	α1	β1	α2	β2	α1	β1	α2	β2
**HisE7 NE2**	858	3169	5403	7713	857	3170	5402	7715
**Heme Fe**	2140	4473	6685	9018	2141	4474	6687	9020
**HisF8 NE2**	1273	3612	5818	8156	1274	3613	5820	8158
**Asp(α_1G1) 94 CG**	1393				1394			
**Tyr(α_1C7) 42 CZ**	616				616			
**Arg(α_1FG4) 92 CD**	1356				1357			
**Thr(α_1C6) 41 OG**	596				596			
**Asn(β_2G4) 101 ND2**				8309				8311
**Arg(β_2C6) 39 CZ**				7366				7368
**Arg(β_2C6) 39 CB**				7355				7357
**Heme NA**	2141		6686		2142		6688	
**Heme NB**	2142		6687		2143		6689	
**Heme NC**	2143		6688		2144		6690	
**Heme ND**	2144		6689		2145		6691	

Table S5. Trout IV DHb atom and residue numbers for post processing analysis.

	Trout IV							
	native				α-prot			
	α1	β1	α2	β2	α1	β1	α2	β2
**HisE7 NE2**	926	3296	5602	7972	924	3297	5601	7974
**Heme Fe**	2267	4604	6943	9280	2268	4605	6945	9282
**His F8NE2**	1370	3752	6046	8428	1371	3753	6048	8430
**Asp(α_1G1) 95 CG**	1487				1488			
**Tyr(α_1C7) 42 CZ**	670				670			
**Arg(α_1FG4) 93 CD**	1450				1451			
**Ala(α_1C6) 41**								
**Asn(β_2G4) 102 ND2**				8583				8585
**Arg(β_2C6) 40 CZ**				7641				7643
**Arg(β_2C6) 40 CB**				7630				7632
**Heme NA**	2268		6944		2269		6946	
**Heme NB**	2269		6945		2270		6947	
**Heme NC**	2270		6946		2271		6948	
**Heme ND**	2271		6947		2272		6949	

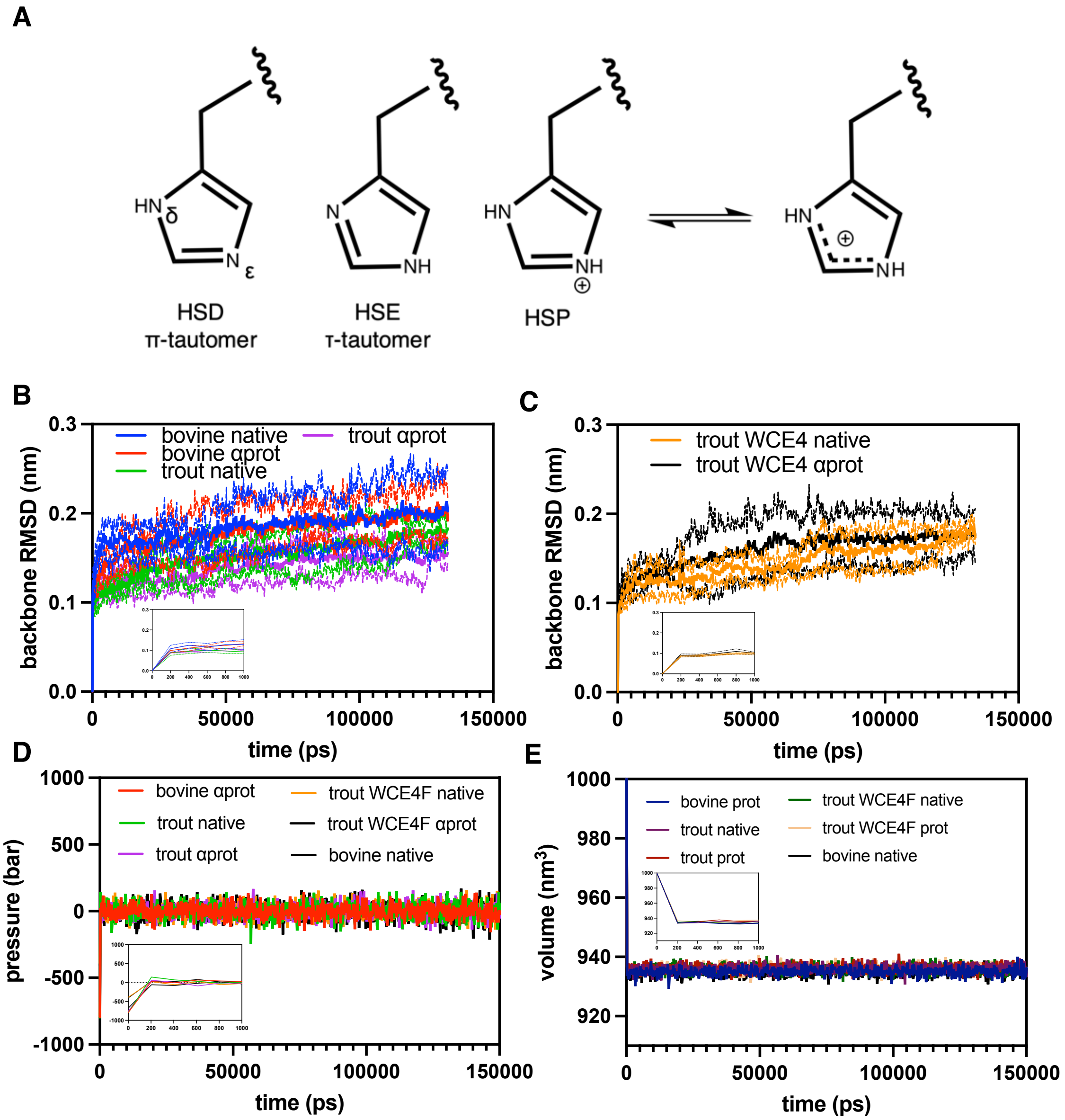

Figure S1. (A) Histidine naming conventions. (B) Average backbone RMSD for bovine and trout IV DHb in their α-chain HisE7 protonated and deprotonated state. (C) Average backbone RMSD for trout WCE4 mutant IV DHb in their α-chain HisE7 protonated and deprotonated state. (D) Average MD simulation pressure. (E) Average MD simulation volume. (B-C) Dashed lines represent standard deviations.

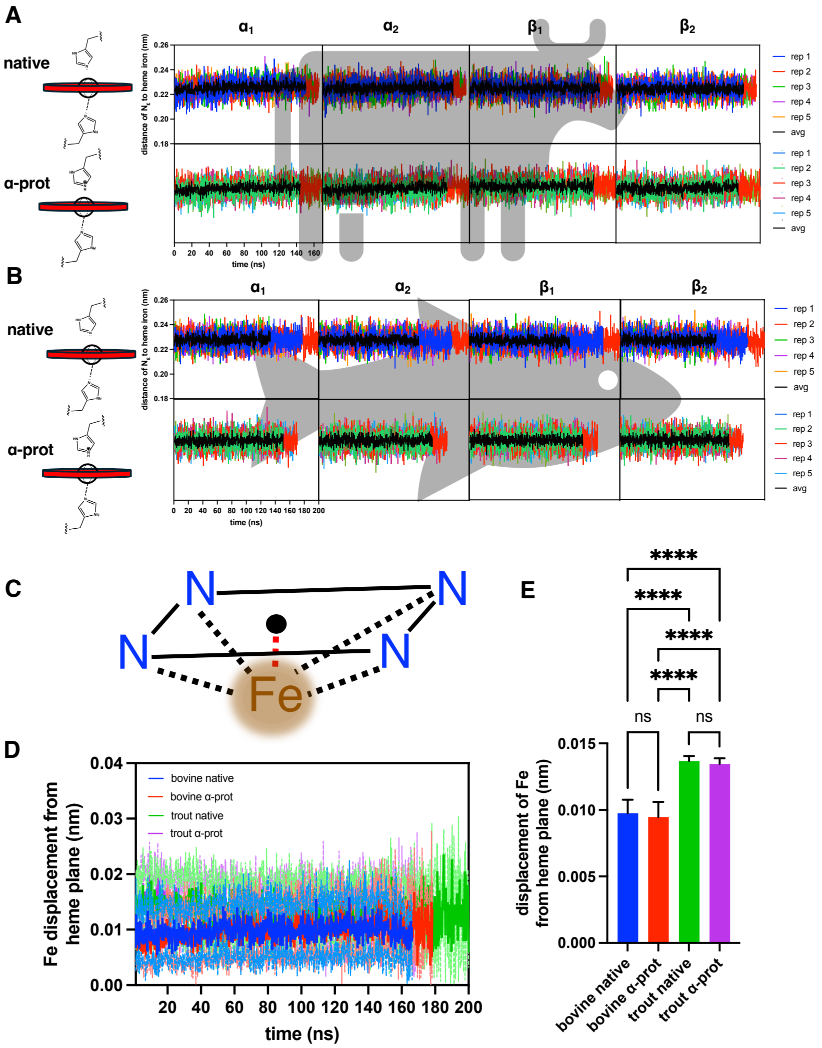

Figure S2. Heme iron distance fo HisF8 Nε and displacement from heme plane. (A) Bovine deoxyhemoglobin (DHb), (top row) distance between HisF8 N_ε and heme iron for all chains, (bottom row) distance between HisF8 N_ε and heme iron when the HisE7 of the alpha chains was in its protonated state, and the beta HisF8 remained deprotonated. (B) Trout IV DHb, (top row) distance between HisF8 N_ε and heme iron for all chains, (bottom row) distance between HisF8 N_ε and heme iron when the HisE7 of the alpha chains was in its protonated state, and the beta HisF8 remained deprotonated. (C) Schematic of heme iron displacement from the heme plane. Analysis was performed by indexing all four-pyrrole nitrogen's then measuring distance (red dashed line) between the nitrogen center or mass (black dot) and the iron atom. (D) α-chain heme iron displacement over the course of the simulation. Data are represented as the average and standard deviation across 5 simulations each with 2 α-chains (n=10 per treatment). (E) α-chain heme iron displacement. Data are represented as the average and standard deviation of all data points between 1-133 ns for all simulations (6,610 data points per group).

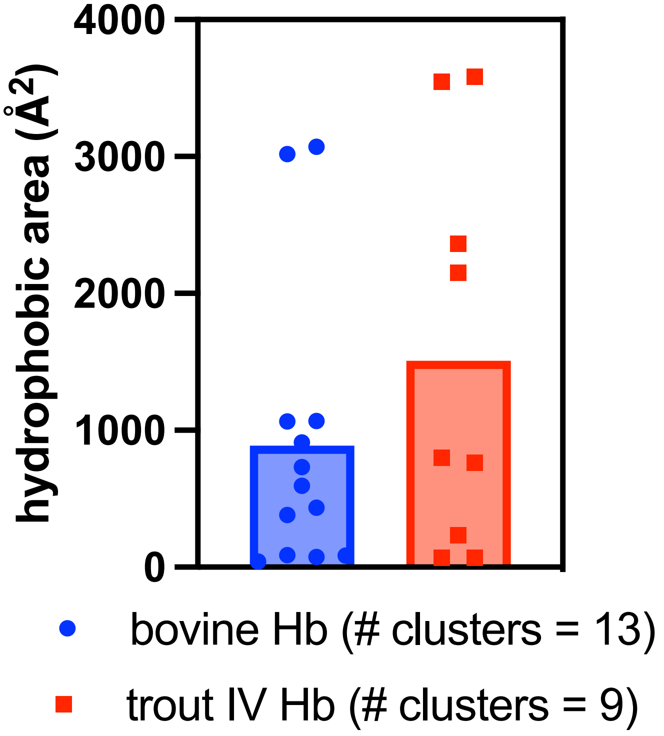

Figure S3. Calculated hydrophobic surface area using ProteinTools[1].

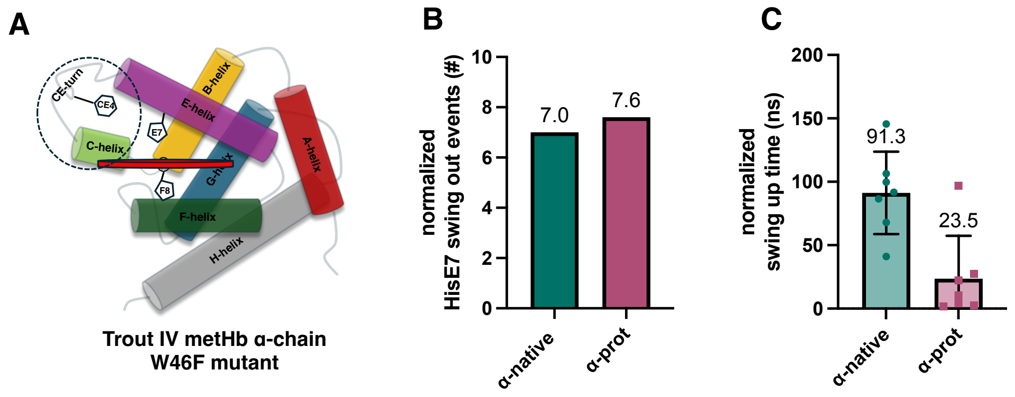

Figure S4. HisE7 swing out events and normalized simulation time of the E7 histidine to swing away from the heme pocket of native alpha (α-native) and beta (β-native) trout IV deoxyhemoglobin (DHb) W46F mutant, and alpha chain with a protonated E7 histidine (α-prot). (A) Location of the CE4 (res. 46) (B) normalized HisE7 swing out events (C) normalized simulation time for HisE7 to swing out.

Output Excerpt from step2_drude.out:

```
Comparing "NONE" and "CNEU".
IF test evaluated as false.  Skipping command

CHARMM>       if @cterpatch .eq. CT1  if @cname .eq. GLY set cterpatch CT1G
Parameter: CTERPATCH -> "NONE"
Parameter: CNAME -> "HEME"
Comparing "NONE" and "CT1".
IF test evaluated as false.  Skipping command

CHARMM>       if @cterpatch .eq. CT2  if @cname .eq. GLY set cterpatch CT2G
Parameter: CTERPATCH -> "NONE"
Parameter: CNAME -> "HEME"
Comparing "NONE" and "CT2".
IF test evaluated as false.  Skipping command

CHARMM>

CHARMM>       set gene = 0
Parameter: GENE <- "0"

CHARMM>       if @type eq protein then
Parameter: TYPE -> "ELSE"
Comparing "ELSE" and "PROTEIN".
IF test evaluated as false.  Skip to ELSE or ENDIF

CHARMM>

CHARMM>       if @type eq rna then  !UMB: explicitly for rna
Parameter: TYPE -> "ELSE"
Comparing "ELSE" and "RNA".
IF test evaluated as false.  Skip to ELSE or ENDIF

CHARMM>

CHARMM>       if @type eq dna then     !UMB: explicitly for dna
Parameter: TYPE -> "ELSE"
Comparing "ELSE" and "DNA".
IF test evaluated as false.  Skip to ELSE or ENDIF

CHARMM>

CHARMM>       if @type eq carb then
Parameter: TYPE -> "ELSE"
Comparing "ELSE" and "CARB".
IF test evaluated as false.  Skip to ELSE or ENDIF

CHARMM>

CHARMM>       if gene .eq. 0 then
Comparing "0" and "0".
IF test evaluated as true.  Performing command

CHARMM>          ! default generate if not protein or water

CHARMM>          generate @segname first none last none setup warn drude dmass 0.4 ! show
Parameter: SEGNAME -> "HETA"
Drude polarizability will be setup for SEGID: HETA    mass of Drudes particles =    0.4000

***** ERROR in GENIC ***** Residue 'HEME  ' was not found.
```

Figure S5. Failure of *Drude prepper* to categorize HEME moiety.

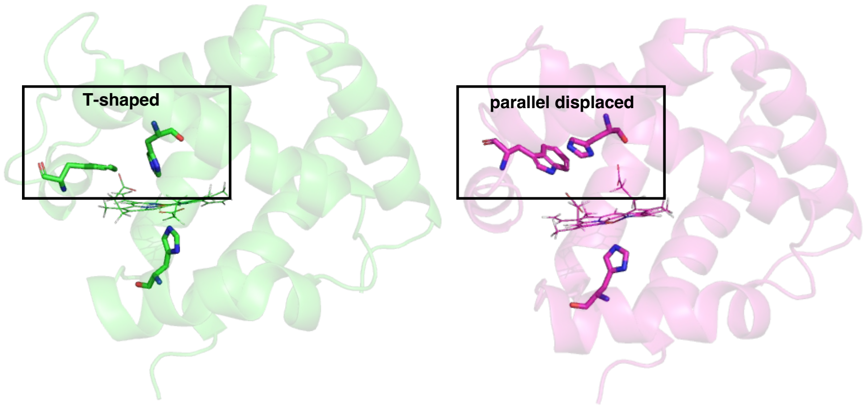

Figure S6. Visualization of T-shaped and parallel displaced π-π stacking interactions.

Equation S1[2]

$$U(\vec{r}) = \sum U_{bonded}(\vec{r}) + \sum U_{non-bonded}(\vec{r})$$

Equation S2[2]

Bond Length | Bond Angle | Improper Dihedral (Out-of-plane Bending) | Dihedrals | Urey - Bradley Term | Backbone correction map

$$U_{bonded}(\vec{r}) = \sum_{\substack{bond\\length}} K_b(b-b_0)^2 + \sum_{\substack{valence\\angle}} K_\theta(\theta-\theta_0)^2 + \sum_{\substack{improper\\dihedrals}} K_\psi(\psi-\psi_0)^2 + \sum_{dihedrals}\sum_{n=1}^{6} K_{\phi,n}\,(1+\cos(n\,\phi-\delta_n)) + E_{UB} + E_{CMAP}$$

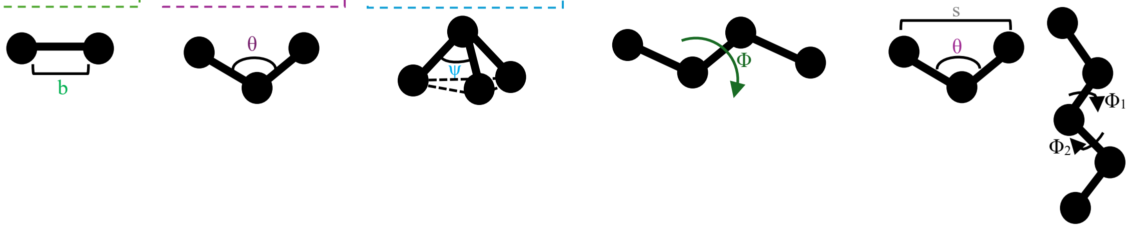

Equation S3[2]

Electrostatic Interactions | Van der Waals Interactions (LJ Potential)

$$U_{non-bonded}(\vec{r}) = \sum_{\substack{electrostatic\\non-bonded\\pairs\ i,j}} \frac{q_i q_j}{4\pi D\|\vec{r_i}-\vec{r_j}\|} + \varepsilon_{ij}\sum_{\substack{Van\ der\ Waals\\non-bonded\\pairs\ i,j}} \left[\left(\frac{R_{min,ij}}{\|\vec{r_i}-\vec{r_j}\|}\right)^{12} - 2\left(\frac{R_{min,ij}}{\|\vec{r_i}-\vec{r_j}\|}\right)^{6}\right]$$

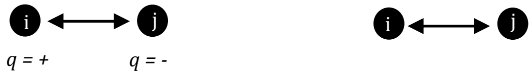

Supporting References

(1) Ferruz, N., Schmidt, S., & Höcker, B. ProteinTools: a toolkit to analyze protein structures. *Nucleic Acids Res.* **2021**, *49*(W1), W559-W566.

(2) Vanommeslaeghe, K., & MacKerell Jr, A. D. CHARMM additive and polarizable force fields for biophysics and computer-aided drug design. *Biochim. Biophys. Acta, Gen. Subj.* **2015**, *1850*(5), 861-871.
